# T Cell Activation Induces Synthesis of CD47 Proteoglycan Isoforms and Their Release in Extracellular Vesicles

**DOI:** 10.3390/ijms26178377

**Published:** 2025-08-28

**Authors:** Sukhbir Kaur, Svetlana A. Kuznetsova, John M. Sipes, Satya P. Singh, Rafael Villasmil, David D. Roberts

**Affiliations:** 1 Laboratory of Pathology, Center for Cancer Research, National Cancer Institute, National Institutes of Health, Bethesda, MD 20982, USA; kuznetsova4872@yahoo.co.uk (S.A.K.); sipesj@mail.nih.gov (J.M.S.); 2 HIV and AIDS Malignancy Branch, Center for Cancer Research, National Cancer Institute, National Institutes of Health, Bethesda, MD 20982, USA; 3 Inflammation Biology Section, Laboratory of Molecular Immunology, National Institute of Allergy and Infectious Diseases, National Institutes of Health, Bethesda, MD 20892, USA; spsingh@niaid.nih.gov; 4 Flow Cytometry Core Facility, National Eye Institute, Bethesda, MD 20892, USA; villasmilr@nei.nih.gov

**Keywords:** T lymphocyte activation, extracellular vesicles, CD47, proteoglycan, thrombospondin-1, B lymphocyte activation

## Abstract

Thrombospondin-1 potently inhibits T cell activation by engaging its cell surface receptor CD47. This inhibitory signal requires glycosaminoglycan modification of CD47. CD47 also regulates the composition of RNAs in extracellular vesicles released by T cells and their functional activities. Because CD47 is also present in extracellular vesicles, we examined the effect of T cell activation on CD47 glycoforms in T cells and extracellular vesicles released by these cells. Activation increased both heparan and chondroitin sulfate biosynthesis by globally inducing mRNA levels of the respective glycosaminoglycan synthases and sulfotransferases. T cell activation in the presence of thrombospondin-1 inhibited induction of these biosynthetic enzymes, but not in cells lacking CD47. Therefore, CD47 signaling controls its own post-translational modification by glycosaminoglycans that are required for thrombospondin-1 signaling. Activation of Jurkat T lymphoblasts and primary CD4 and CD8 T cells increased the release of proteoglycan isoforms of CD47 and amyloid precursor-like protein-2 associated with extracellular vesicles and smaller macromolecular complexes. However, cell surface levels of CD47 were minimally changed during activation. BJAB and RAJI B cell lines also produced CD47^+^ extracellular vesicles and showed increased release of highly glycosylated CD47 following B cell receptor engagement. Therefore, T and B lymphocyte activation results in a selective increase in the synthesis and release of extracellular vesicles containing proteoglycan isoforms of CD47.

## 1. Introduction

Glycosaminoglycans are polymeric repeating disaccharides that are covalently attached via core O-linked oligosaccharides to specific serine residues in core proteins and are present throughout the animal kingdom [1]. Glycosaminoglycan chains contain alternating glucuronic acid and N-acetylglucosamine or N-acetylgalactosamine residues, giving rise to heparan and chondroitin sulfates, respectively [2,3,4,5,6]. These polysaccharides are further modified by specific patterns of sulfation, glucuronic acid epimerization, and N-acetylglucosamine deacetylation, which determine their interactions with ligands and co-receptors [7,8,9].

Heparan sulfate proteoglycans (HSPGs) regulate growth and development by playing a central role linking the extracellular matrix and intracellular signaling; this can influence cell fate and differentiation and bone repair and tissue regeneration by mediating interactions with growth factors, tyrosine kinases, integrins, chemokines, and cytokines [10,11,12,13]. HSPGs also play roles in inflammation [14,15,16] and the immune response to pathogens [17].

Members of the syndecan family and the CD44 variant CD44v3 are the best characterized cell surface proteoglycans expressed by T cells [18,19,20]. Expansion of TCRβ+ T cells expressing syndecan-1 (CD138) correlated with disease severity in lupus-prone MRL/Lpr mice [21,22]. Syndecan-1 expressed on gamma/delta T cells regulated their homeostasis and pathogenesis of psoriasis-like skin inflammation [19]. Loss of syndecan-1 on T cells limited their proliferative responses [23]. Expression of CD44v3 was increased on T cells from lupus patients with active disease [24].

Syndecans and glypicans have also been widely studied for their role as an attachment receptor for viruses [25,26]. Cooperation between viral and host signaling leads to spread and establishment of viral infection (including GP120 interactions with HSPG [27]). HIV1 p17 induced inflammatory cytokine responses by interacting with heparan sulfate modified syndecan-2, syndecan-4, and the CD44 variant CD44v3 [20].

HIV-1 Tat and HSPGs regulate the activation of lymphocyte trans-endothelial cell migration [28]. Viral particles and extracellular vesicles share common processes in biogenesis, and heparan sulfate proteoglycans regulate the composition, uptake, and secretion of extracellular vesicles (EVs) [29,30,31,32]. Syndecan-1 is present on plasma-derived EVs from glioma patients [33]. Therefore, HSPGs are an attractive target to block viral entry or therapy and EV functions in cell–cell communication.

CD47 is a cell surface transmembrane protein that functions as a counter receptor for signal regulatory protein-α (SIRPα) on monocytes in the immune system. The resulting SIRPα signaling limits the removal of CD47-expressing cells by phagocytes and antigen presentation to T cells by dendritic cells [34,35,36]. The CD47 expressed on vascular and immune cells also functions as an inhibitory signaling receptor upon engaging its secreted ligand thrombospondin-1 [37,38]. Thrombospondin-1 binding to CD47 on T cells inhibits their activation [38,39,40,41,42,43,44]. Conversely, blocking this CD47 signaling on mouse and human cytotoxic CD8 T cells increases their antigen-dependent killing of cancer cell targets [45,46,47]. CD47 mRNA expression was increased in activated human CD4 and CD8 T cells and associated with high expression of markers of T-cell cytotoxicity and exhaustion [48].

Heparan sulfate modification of CD47 at Ser^64^ is necessary for the ability of thrombospondin-1 to inhibit T-cell activation [42]. Heparan sulfate-modified CD47 similarly mediates inhibition of T-cell activation by Blastomyces adhesin-1, which shares a heparin binding motif with thrombospondin-1 [49]. Metabolic labeling studies indicated that proteoglycan isoforms of CD47 are released into the medium by Jurkat T lymphoblast cells and by unstimulated primary CD4 and CD8 T cells [42]. The mechanism by which membrane-bound CD47 is released from T cells is unclear, but may include proteolytic cleavage of its extracellular IgV domain from the transmembrane domain, which has been demonstrated in other cell types [50,51,52].

Subsequently, we found that intact CD47 is released in extracellular vesicles (EVs) produced by Jurkat cells and regulates the packaging of specific RNAs into these EVs [53,54,55]. These EVs have CD47-dependent effects on gene expression when taken up by target cells [53,56]. This prompted us to examine whether T-cell activation alters the release of CD47 and its proteoglycan glycoform into EVs and the regulation of this process by thrombospondin-1. Here, we examined how activation alters the biosynthesis of CD47 glycoforms by primary human T cells and Jurkat T lymphoblasts and the association of the released CD47 with EVs. These studies indicate that T cell receptor signaling regulates the glycosaminoglycan modification of CD47 and stimulates the release of these CD47 isoforms in EVs. Activation-induced release of CD47 associated with EVs was also observed in two human B cell lines.

## 2. Results

### 2.1. Activation- and Thrombospondin-1-Dependent Regulation of T-Cell Proteoglycan Biosynthesis

**Figure 1:**
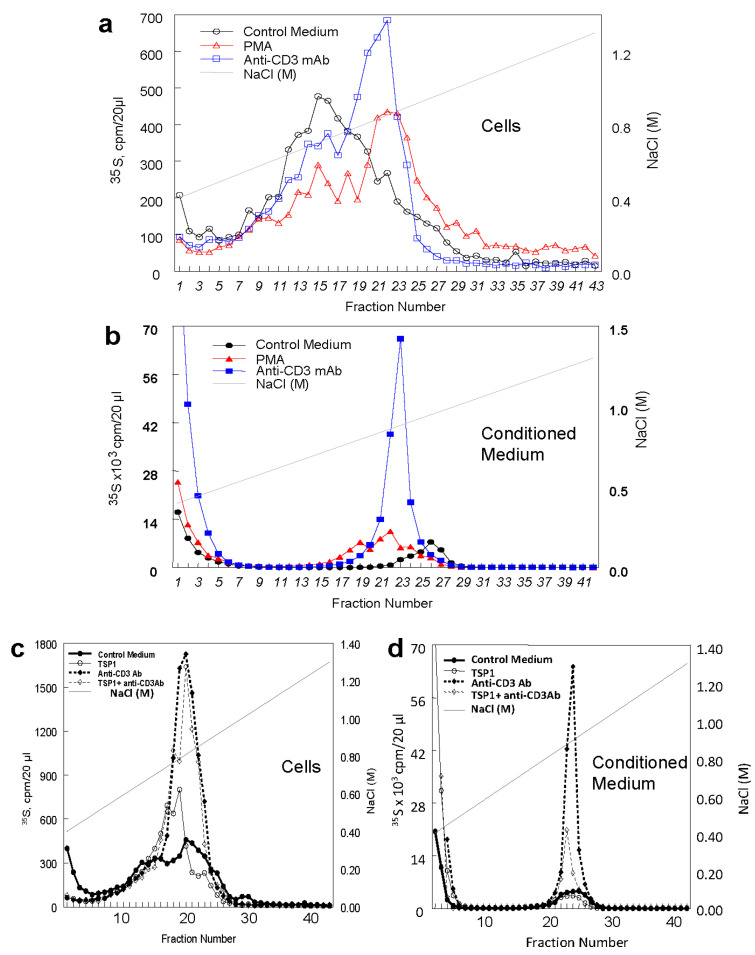
Induction of proteoglycan biosynthesis in Jurkat T cells via CD3 antibody and phorbol ester. Ion exchange analysis of cell (**a**) and medium proteoglycans (**b**) was performed by gradient elution from Q Sepharose columns after metabolic labeling of resting cells (○, ●), cells plated on immobilized CD3 antibody (□, ■), and cells labeled in the presence of 1 µM PMA (Δ, ▲). (**c**,**d**) Thrombospondin-1 inhibits anti-CD3-induced proteoglycan synthesis and release. Jurkat cells were metabolically labeled without treatment (●), while plated on immobilized anti-CD3 (1 µg/mL) (♦), plated on immobilized TSP1 (20 µg/mL) (○), or plated on immobilized anti-CD3 and thrombospondin-1 (◊). Incorporation of ^35^S-sulfate in proteoglycans was analyzed by ion exchange on Q Sepharose with NaCl gradient elution.

Metabolic labeling with ^35^S-sulfate followed by ion-exchange chromatography demonstrated an increase in more highly charged proteoglycans isolated from Jurkat cells when activated by plating on immobilized CD3 antibody and a lesser increase when the cells were activated by incubation with the protein kinase C activator phorbol 12-myristate 13-acetate (PMA) (Figure 1a). However, a more dramatic increase in ^35^S-sulfate incorporation was seen in the conditioned medium from cells activated on immobilized CD3 antibody relative to conditioned medium from unstimulated cells or cells stimulated with PMA (Figure 1b).

**Figure 2:**
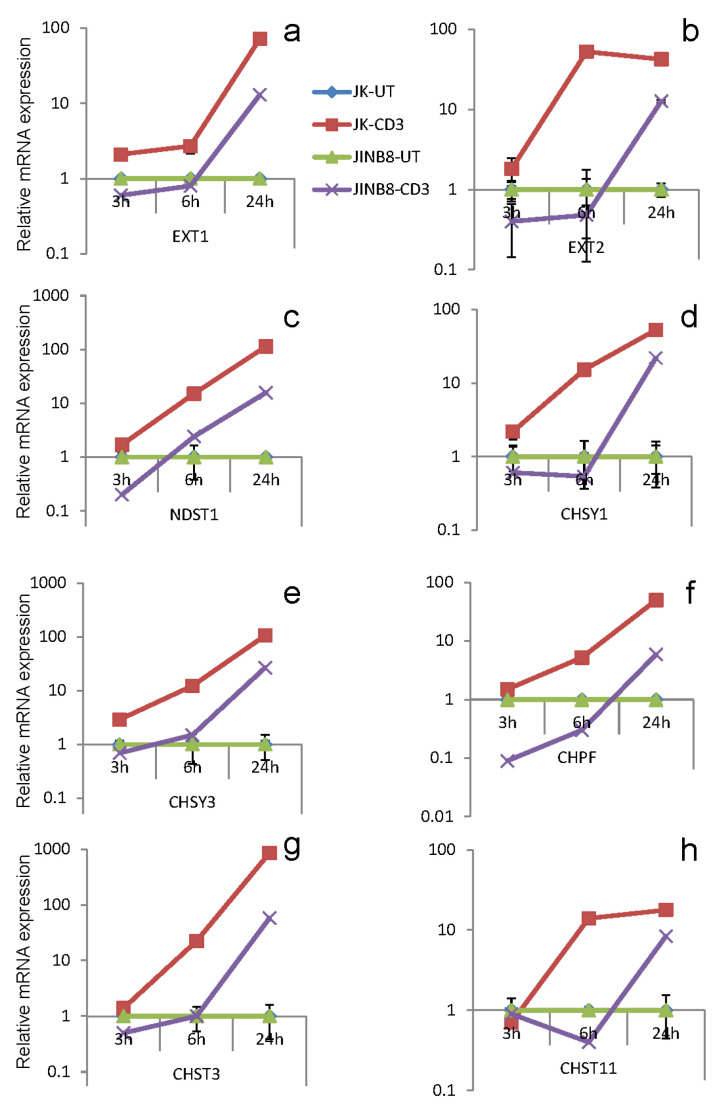
Jurkat T lymphoblast activation induces mRNAs encoding biosynthetic enzymes for heparan and chondroitin sulfates. (**a**–**h**) WT (♦, ■) and CD47-deficient Jurkat T cells (▲, x) were plated on uncoated wells (♦, ▲) or wells coated with anti-CD3 antibody (■, x) and incubated for the indicated times. RNA isolated from the cells was analyzed by real-time PCR using primers for the indicated mRNAs and β2M for normalization. Fold-changes in mRNA were calculated by the ΔΔCt method and are presented normalized to initial levels in the respective untreated cells. Results are mean ± SD.

Consistent with the increased glycosaminoglycan biosynthesis following activation, the levels of several mRNAs encoding enzymes mediating heparan and chondroitin chain polymerization and sulfation were dramatically induced over the 24 h following plating Jurkat cells on immobilized anti-CD3 (Figure 2). These included EXT1 and EXT2, which mediate heparan sulfate chain elongation and the heparan N-deacetylase and N-sulfotransferase NDST2 [57,58]. Transcripts encoding several enzymes involved in chondroitin sulfate biosynthesis were strongly induced during Jurkat cell activation, including the chondroitin synthases CHSY1 and CHSY3, the polymerizing factor CHPF, and the sulfotransferases CHST3 and CHST11 [59]. Consistent with the impaired anti-CD3 activation reported for the CD47-deficient mutant JinB8 [60], induction of mRNAs for the same biosynthetic enzymes was delayed or reduced in this Jurkat cell mutant lacking CD47.

Jurkat cells are widely used to study signal transduction pathways involved in T cell activation, but some pathways diverge in this transformed cell line. We examined previous RNA sequencing data to determine whether glycosaminoglycan biosynthesis pathways are similarly induced during the activation of primary T cells. Consistent with the Jurkat cell results, expression of CHSY1, CHPF, and CHST11 mRNAs was significantly induced following 6 h activation of CD8 T cells from mouse spleen using CD3 and CD28 antibodies (Table 1, based on Data Supplement S1 in [61]). In addition, mRNAs encoding the heparan-chain-initiating GlcNAc transferase EXTL2 and the D-glucuronyl C5-epimerase (GLCE) were significantly induced at 6 h, with decreased induction observed in activated Cd47^−/−^ CD8 T cells (Table 1).

**Figure 3:**
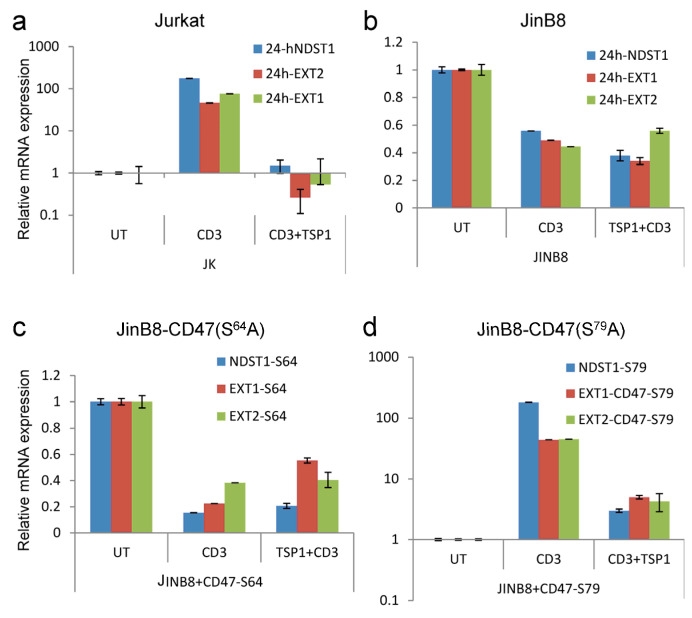
Thrombospondin-1 inhibits anti-CD3-induced expression of mRNAs encoding NDST1, EXT1, and EXT2 in a CD47-dependent manner. A-D, Jurkat T cells (**a**), the CD47-deficient Jurkat mutant JinB8 (**b**), or JinB8 transfected with plasmids to express thrombospondin-1-insensitive CD47(S^64^A) mutant (**c**) or thrombospondin-1-sensitive CD47(S^79^A) mutant (**d**) were plated on immobilized anti-CD3 (1 µg/mL) for 6 h in the absence or presence of 1 µg/mL thrombospondin-1, and relative mRNA levels were quantified by real-time PCR.

The CD47 ligand thrombospondin-1 inhibits T cell activation and the expression of activation-dependent genes in T cells [39,42,43,44]. Similar inhibition was observed for the anti-CD3-induced ^35^S-proteoglycans released in Jurkat cell-conditioned medium (Figure 1c,d). Correspondingly, the induction of EXT1, EXT2, and NDST1 mRNAs by anti-CD3 activation was inhibited in the presence of thrombospondin-1 (Figure 3a). Consistent with the reported impaired anti-CD3 activation of the CD47-deficient mutant JinB8 cells [60], mRNAs for the same biosynthetic enzymes were not induced in these cells (Figure 3b) or JinB8 transfected with a nonsignaling CD47(S^64^A) mutant (Figure 3c); however, re-expressing the functional heparan sulfate-modified CD47 mutant S^79^A restored sensitivity to thrombospondin-1 inhibition (Figure 3d).

Double-labeling of Jurkat cells using ^3^H-glucosamine and ^35^S-sulfate combined with enzymatic digestion was used to further characterize the glycosaminoglycans released into the medium upon Jurkat cell activation (Table 2). Anti-CD3 stimulation dramatically increased glucosamine as well as sulfate incorporation into glycosaminoglycans (9.2-fold and 6.5-fold, respectively). However, the ratio of ^35^S/^3^H decreased by 32% following activation, indicating that the average degree of sulfation decreases upon T cell activation. The fraction of ^3^H-glucosamine incorporated into proteoglycan in the medium that was resistant to chondroitinase ABC digestion decreased from 38% in unstimulated cells to 25% after anti-CD3 stimulation.

### 2.2. T Cell Activation Induces Release of Proteoglycan Isoforms of CD47 and APLP2

**Figure 4:**
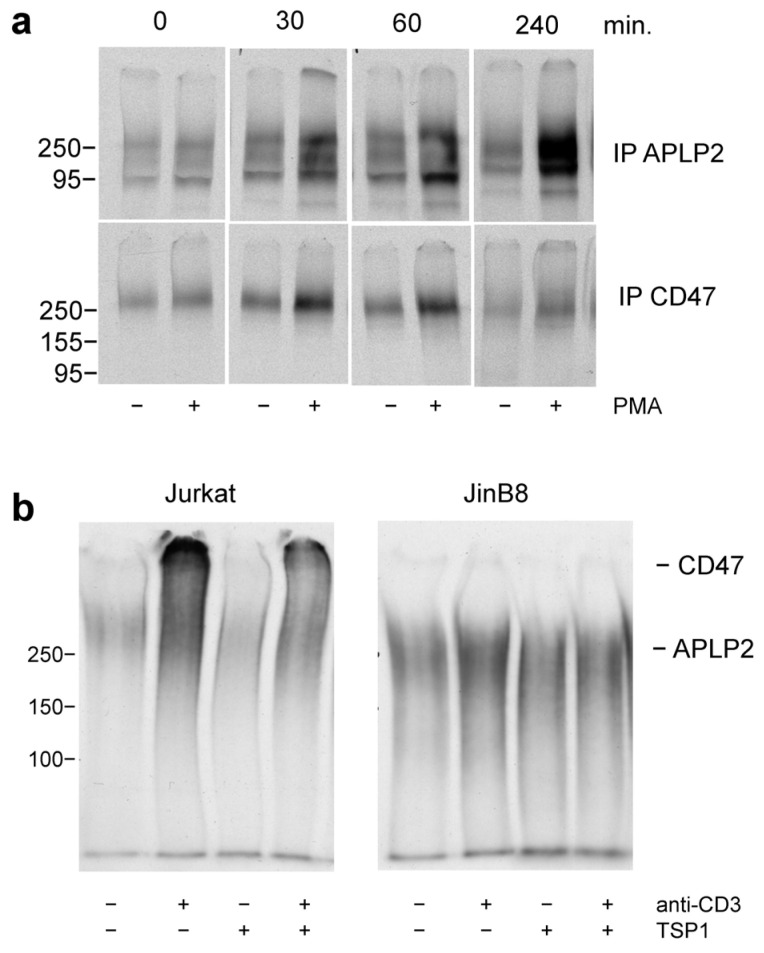
(**a**) Induction of proteoglycan isoforms of CD47 and APLP2 in Jurkat T cell-conditioned medium by PMA. Jurkat cells were labelled in medium with ^35^S-sulfate and stimulated using 1 µM PMA for 0 to 240 min. Pooled proteoglycan fractions in the conditioned medium were isolated by ion exchange, immunoprecipitated using APLP2 and CD47 antibodies, and subjected to SDS gel electrophoresis on 7.5% acrylamide SDS gels. ^35^S-labeled isoforms of CD47 and APLP2 were visualized by fluorography. (**b**) Thrombospondin-1 (TSP1) inhibits the induction of proteoglycan isoforms of CD47 and APLP2 released following activation by immobilized anti-CD3. Wild-type Jurkat cells and CD47-deficient mutant JinB8 cells were labelled with ^35^S-sulfate and activated on immobilized anti-CD3 for 24 h in the absence or presence of 2 µg/mL TSP1. Pooled proteoglycan fractions were isolated from conditioned medium by ion exchange and subjected to SDS gel electrophoresis on a 5% acrylamide gel. ^35^S-labeled isoforms of CD47 and APLP2 were visualized by fluorography.

Our previous studies identified amyloid precursor-like protein-2 (APLP2) and CD47 as the major proteoglycans expressed by unstimulated Jurkat cells [42]. To identify which protein cores are responsible for the increased proteoglycan release observed following activation, conditioned medium from PMA-stimulated Jurkat cells was immunoprecipitated using APLP2 and CD47 antibodies. SDS gel electrophoresis of the immunoprecipitates showed maximal induction of CD47 at 30–60 min., whereas APLP2 induction was highest at 4 h (Figure 4a). As reported previously, the proteoglycan isoform of CD47 migrated with a higher apparent molecular weight than the labeled APLP2 isoforms.

As expected, the high-molecular-weight proteoglycan was absent in the conditioned medium from activated CD47-deficient JinB8 cells (Figure 4b, right panel). CD47 induction was decreased when Jurkat cells were activated in the presence of thrombospondin-1, suggesting that thrombospondin-1 limits the expression or extracellular release of its receptor during T cell activation (Figure 4b, left panel). A lesser induction of the proteoglycan isoforms of APLP2 was visible in the anti-CD3-activated JinB8 cell medium, indicating that CD47 is the major core protein responsible for the increased proteoglycan release observed using anti-CD3 activation.

**Figure 5:**
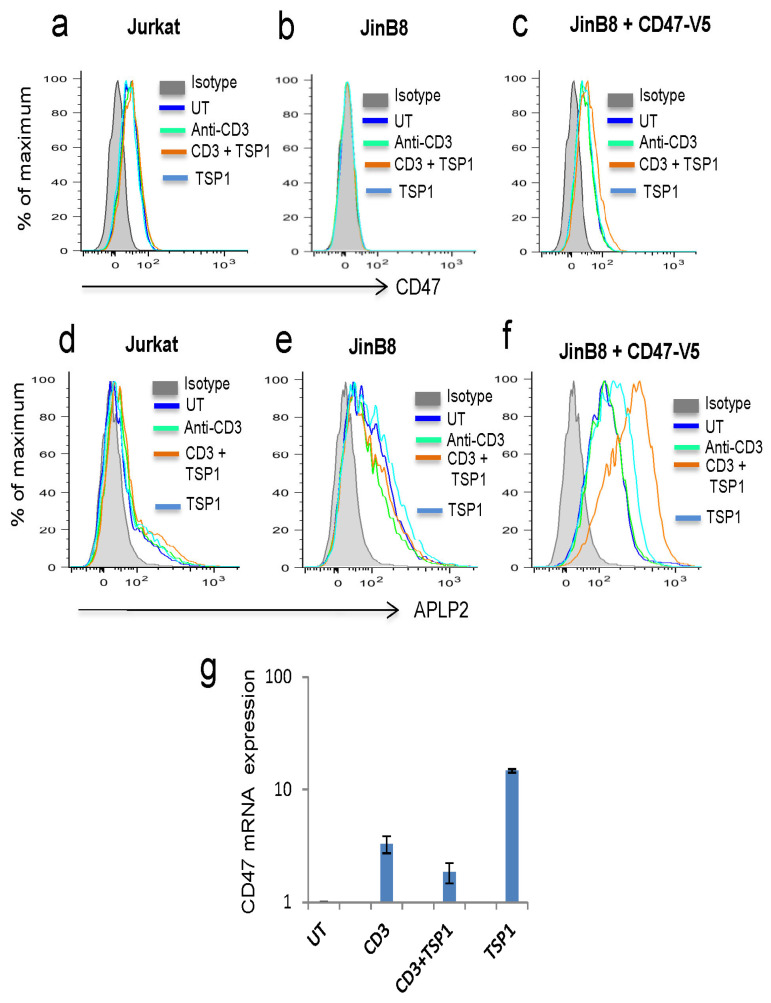
Effects of activation and thrombospondin-1 on cell surface CD47 and APLP2 expression. (**a**) Flow cytometry analysis of cell surface CD47 expression in unstimulated wild-type Jurkat cells and following stimulation on immobilized anti-CD3 in the presence or absence of 1 µg/mL thrombospondin-1. (**b**,**c**) Analysis of cell surface CD47 on CD47-deficient JinB8 cells and the same cells following transient re-expression of CD47 following activation and thrombospondin-1 treatments. (**d**–**f**) Analysis of cell surface APLP2 levels on the same cells following activation with or without thrombospondin-1 treatment. (**g**) Effects of anti-CD3 activation and thrombospondin-1 treatment on CD47 mRNA expression in Jurkat cells.

The results in Figure 1a suggest that activation increases the release of CD47 into the medium, while cellular levels are generally maintained. Flow cytometry confirmed that cell surface CD47 levels are minimally altered following anti-CD3 activation of Jurkat cells (Figure 5a). Thrombospondin-1 treatment alone or combined with activation also had minimal effects on cell surface CD47. As expected, JinB8 cells lack cell surface CD47, and re-expressing CD47 restored cell surface levels that remained relatively insensitive to activation or thrombospondin-1 treatments (Figure 5b,c).

We compared the effects of activation and thrombospondin-1 on cell surface APLP2, which, based on Figure 4, is also released from Jurkat cells following activation. Flow cytometry indicated more heterogeneity in the cell surface expression of APLP2 (Figure 5d). Cell surface APLP2 was relatively insensitive to activation, but activation in the presence of thrombospondin-1 led to some increase in the fraction of Jurkat cells with higher APLP2 levels. Cell surface APLP2 was basally higher in CD47-deficient JinB8 cells and minimally affected by activation or thrombospondin-1 treatment (Figure 5e). However, cell surface APLP2 was further elevated when CD47 was re-expressed in these cells, and thrombospondin-1 treatment further enhanced cell surface APLP2 (Figure 5e,f).

Maintaining constant cell surface CD47 levels while increasing its release from the cells suggested that T cell activation is associated with increased CD47 biosynthesis, which was confirmed by an increase in CD47 mRNA when cells were activated on immobilized anti-CD3 (Figure 5g). As expected, thrombospondin-1 treatment limited this induction by anti-CD3 activation, but thrombospondin-1 alone also increased CD47 mRNA.

### 2.3. Activation Stimulates Release of Proteoglycan Isoforms of CD47 and APLP2 by Primary Human T Cells

**Figure 6:**
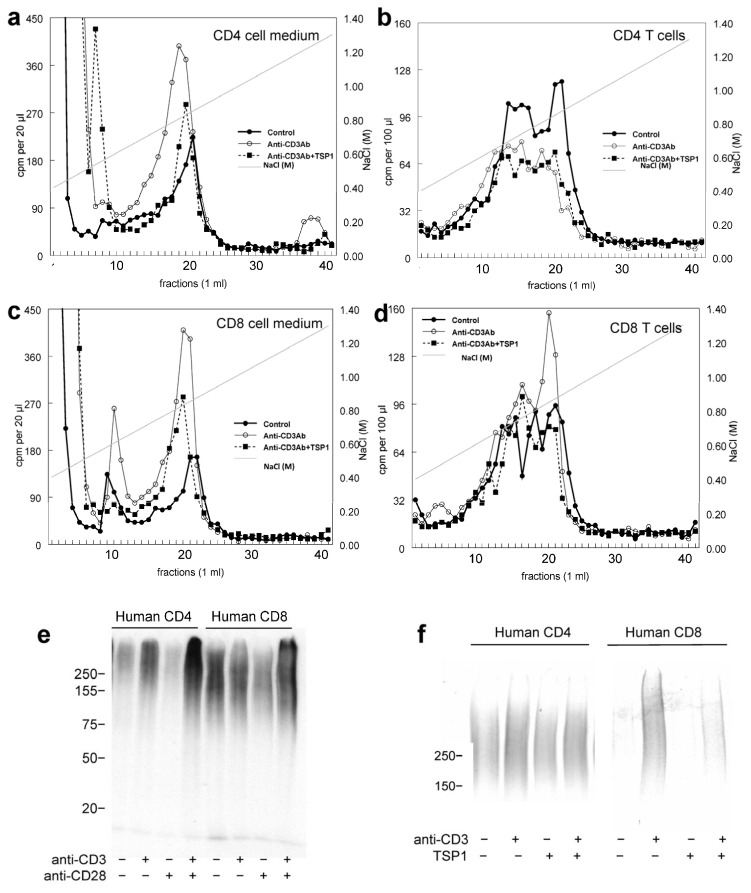
Thrombospondin-1 (TSP1) inhibits activation-induced proteoglycan synthesis and release in human peripheral blood CD4 and CD8 T cells. CD4 (**a**,**b**) and CD8 T cells (**c**,**d**) were isolated from PBMCs from a single donor by negative selection and metabolically labeled with ^35^S-sulfate without treatment (●). The cells were plated on immobilized anti-CD3 (1 µg/mL ○) or immobilized anti-CD3 + TSP1 (■). Proteoglycans extracted from the cells, and conditioned media were analyzed by ion exchange on Q Sepharose. (**e**) Human CD4 and CD8 T cells were metabolically labeled with ^35^S-sulfate. Cells were activated by plating on immobilized anti-CD3 or CD28 alone or in combination. Proteoglycan fractions were isolated from the conditioned media by ion-exchange chromatography, analyzed by SDS gel electrophoresis, and detected by fluorography. (**f**) Metabolically labeled proteoglycan fractions isolated from conditioned medium from resting or anti-CD3-activated CD4 and CD8 T cells were analyzed by SDS gel electrophoresis and detected by fluorography. T cells in (**e**,**f**) were from different anonymous donors and are representative of experiments performed using at least one additional donor.

Increased ^35^S-sulfate-labeled proteoglycan release into the medium following anti-CD3 activation was reproduced using CD4 and CD8 T cells isolated from human peripheral blood (Figure 6a–d). This response was also inhibited when the cells were exposed to anti-CD3 in the presence of thrombospondin-1. As was seen for Jurkat cells, primary CD4 and CD8 T cells released the majority of newly synthesized proteoglycans into the medium over 24 h, and the fold-induction of ^35^S incorporation by anti-CD3 stimulation was greater in the medium fraction than in cells.

SDS gel electrophoresis of the proteoglycan fractions purified from conditioned media from the metabolically labeled CD4 T cells confirmed an increased abundance of heterogeneous high-molecular-weight proteoglycans following activation on anti-CD4 (Figure 6e), which was enhanced by anti-CD28 costimulation. The anti-CD3-induced proteoglycans in conditioned medium were decreased in the presence of thrombospondin-1, with no significant change in apparent size (Figure 6f, left lanes). The proteoglycan in the conditioned media from CD8 T cells showed stronger induction following anti-CD3 activation and was partially inhibited by thrombospondin-1 (Figure 6f, right lanes).

**Figure 7:**
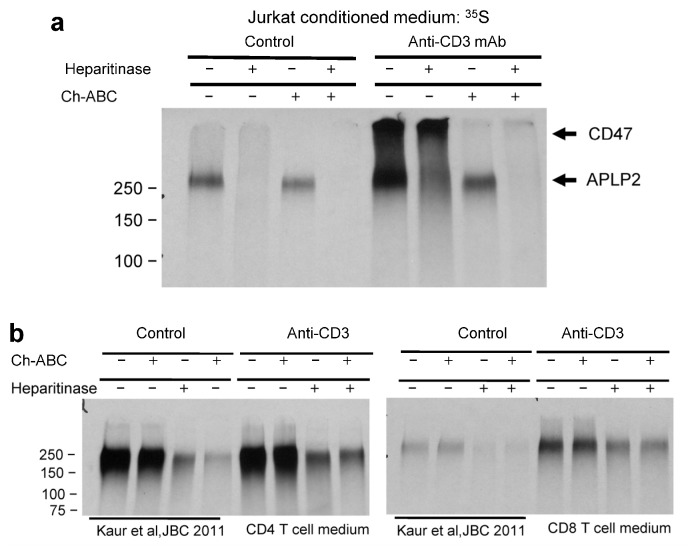
Increased chondroitin sulfate modification of CD47 induced by anti-CD3 stimulation of Jurkat T cells. (**a**) Purified ^35^S-labeled proteoglycan fractions from conditioned media of unstimulated (Control) or anti-CD3-activated Jurkat cells were treated with the indicated combinations of heparitinase or chondroitinase ABC (Ch-ABC) and then analyzed by SDS gel electrophoresis on 7.5% acrylamide SDS gels and detected by fluorography. Migration positions of CD47 and APLP2 are indicated by arrows. (**b**) Proteoglycan fractions purified from metabolically labeled human CD4 and CD8 T cells (data from [42]) and the same cells activated on anti-CD3 were digested with the indicated enzymes and analyzed by SDS gel electrophoresis on 7.5% acrylamide SDS gels and detected by fluorography.

We used glycosaminoglycan-degrading enzymes to examine whether T cell activation could regulate TSP1 signaling through CD47 by altering its glycosaminoglycan modification. Whereas the proteoglycan released by unstimulated Jurkat cells was mostly degraded by heparitinase (Figure 7a left lanes), ^35^S incorporation into the largest proteoglycan, which is mostly CD47, was dramatically increased when Jurkat cells were activated on anti-CD3. The increased labeling of this larger proteoglycan was mostly sensitive to chondroitinase ABC digestion (Figure 7a right lanes). These results also indicate that the APLP2 released by activated Jurkat cells has both heparan sulfate and chondroitin sulfate modifications.

Proteoglycans from the conditioned media of stimulated and unstimulated primary human T cells were purified by ion-exchange chromatography and analyzed by SDS gel electrophoresis after digestion with heparitinase and/or chondroitinase ABC to determine which are induced by T cell activation (Figure 7b). The previously published results for unstimulated Jurkat T cells [42] indicated that most ^35^S incorporation into the high-molecular-weight proteoglycan was sensitive to heparitinase digestion in unstimulated CD4 and CD8 T cells, and most of the remainder in CD4 cells was sensitive to chondroitinase digestion. Following anti-CD3 stimulation, the overall incorporation into the higher-molecular-weight proteoglycan increased more for CD8 T cells than for CD4 T cells, and a larger fraction was more resistant to heparitinase digestion in activated CD4 and CD8 T cells. This indicates a switch towards increased chondroitin sulfate modification of the high-molecular-weight proteoglycan following the activation of primary human T cells.

### 2.4. T Cell Activation Stiumulates the Release of CD47 and APLP2 Glycoforms Associated with EVs

**Figure 8:**
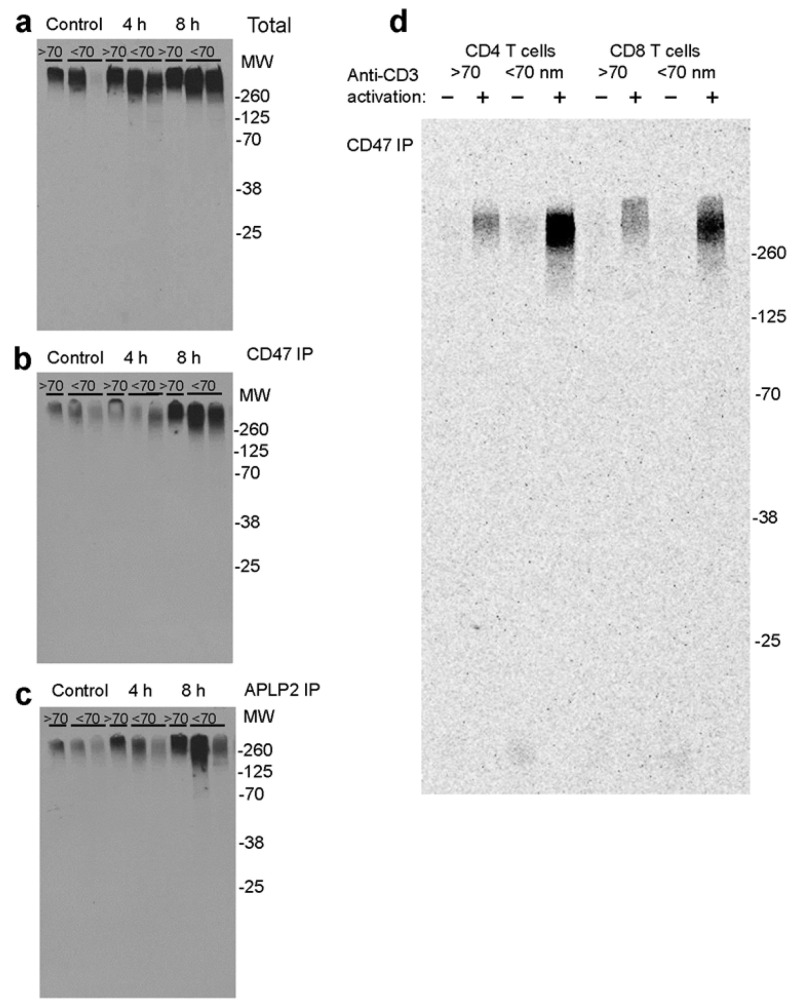
Size analysis of CD47 and APLP2 EVs and smaller particles released by Jurkat and peripheral blood T cells. (**a**–**c**) Jurkat cells were grown overnight in serum-free HITES medium containing ^35^S-sulfate and then plated on immobilized OKT3 anti-CD3 for 4 or 8 h. Then, 40 mL volumes of conditioned media from control and treated cells were concentrated to 0.5 mL by ultrafiltration and applied onto qEV70 columns. Pooled EVs (1) and lower Mr fractions (2,3) were immunoprecipitated using CD47 or APLP2 antibodies. Total and immunoprecipitated fractions were analyzed using 4–12% NuPage Bis-Tris SDS gel electrophoresis and detected by fluorography. (**a**) Total, (**b**) CD47 immunoprecipitation, and (**c**) APLP2 immunoprecipitation. (**d**) Human CD4 and CD8 T cells were labeled overnight using ^35^S-sulfate and then activated on immobilized anti-CD3. The concentrated conditioned media from activated and controls cells were analyzed using qEV70 columns followed by immunoprecipitation using CD47 antibody, SDS gel electrophoresis, and fluorography.

Size-exclusion chromatography was used to determine whether activation of Jurkat cells increases the released proteoglycan isoforms of CD47 and APLP2 that are associated with EVs (Figure 8). ^35^S-proteoglycans were detected in the EV fraction (>70 nm) from the conditioned medium of unstimulated cells, and the levels increased following anti-CD3 activation for 4 or 8 h (Figure 8a). However, activation also increased the high-molecular-weight proteoglycans detected in fractions 2 and 3 corresponding to particle sizes less of than 70 nm, which includes smaller EVs and other macromolecular complexes such as exomeres [62]. Although more isotope was associated with the smaller particles, immunoprecipitation indicated that CD47 and APLP2 were more enriched in the EV fraction from unstimulated cells. Both of these proteins were previously identified in EVs from multiple cell types, including T cells, but their glycosylation states were not determined [53,63,64,65].

Immunoprecipitation of CD47 in the greater than 70 nm EV fractions indicated the strongest induction of CD47 in EVs following 8 h activation (Figure 8b). CD47 was also induced in the less than 70 nm fractions and included lower-molecular-weight forms that could indicate proteolysis. Immunoprecipitation indicated more induction of EVs larger than 70 nm containing APLP2 at 4 h, but the strongest induction was again seen after the 8 h activation (Figure 8c). APLP2 was similarly induced in the less than 70 nm fractions at 8 h and included lower-molecular-weight forms.

Similar size analyses were performed using conditioned medium from metabolically labeled human peripheral blood CD4 and CD8 T cells and the same cells activated on immobilized anti-CD3 + CD28 for 2 h (Figure 8d). Activation strongly induced the release of newly synthesized CD47 from CD4 and CD8 T cells. This included increased CD47 in particles larger than 70 nm characteristic of EVs. Notably, some of the CD47 associated with particles smaller than 70 nm migrated with apparently reduced molecular weight compared to the CD47 in the EV fraction. These smaller particles may contain proteolytically cleaved extracellular domains of CD47 bearing glycosaminoglycan modifications.

### 2.5. B Cell Line Activation Modulates CD47 Glycosylation and Release in EVs

**Figure 9:**
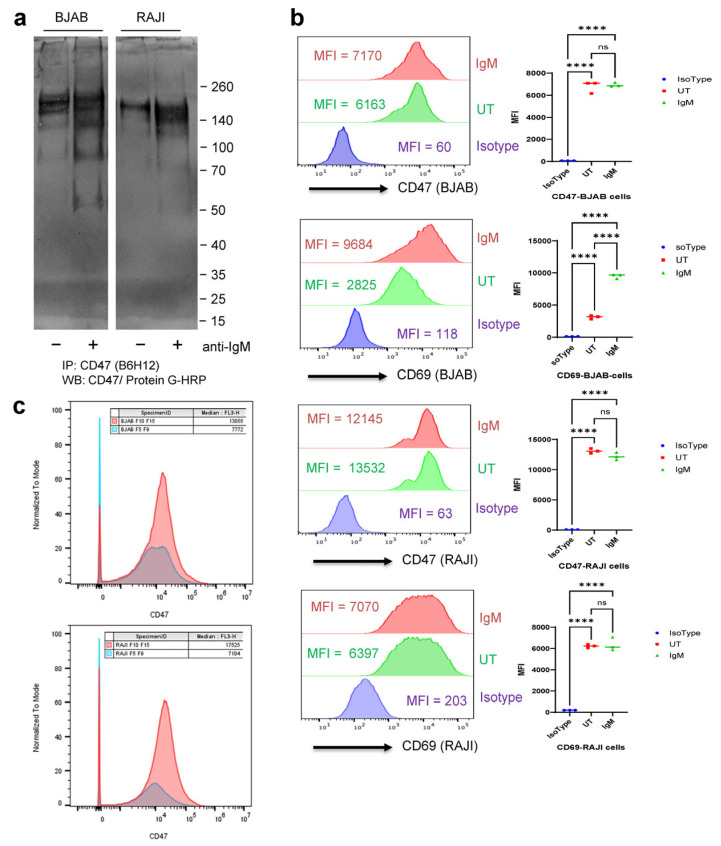
CD47 released by malignant B cells into conditioned media and EVs. (**a**) BJAB and RAJI cells were grown overnight in X-VIVO 15 medium in the presence or absence of 2 µg/mL anti-IgM. Conditioned media from control and treated cells were immunoprecipitated with B6H12 anti-CD47 magnetic beads. (**a**) The immunoprecipitates were analyzed using 4–12% NuPage Bis-Tris SDS gel electrophoresis and detected using HRP-conjugated protein G. (**b**) Expression of CD47 on the cell surface of unstimulated and anti-IgM-activated BJAB cells was analyzed by flow cytometry. (**c**) BJAB and RAJI cells were cultured using X-VIVO media, and 50 mL volumes of conditioned media were concentrated to 0.5 mL by ultrafiltration and applied onto qEV35 columns. Pooled EV fractions (5–9) and fractions (10–15) were immunostained using PE-conjugated CD47 antibody and analyzed via Nanoflow. **** 0.0001.

In addition to the increased CD47 expression in activated human CD4 and CD8 T cells, elevated CD47 mRNA expression in human B cells was associated with high expression of B cell activation markers and antiviral response genes [48]. Three human B cell lines were examined to determine whether the activation of B cells also induces the release of highly glycosylated CD47 associated with EVs. Following activation by engaging the B cell receptor, the immortalized B cell lines BJAB and RAJI exhibited strong increases in thr levels of highly glycosylated CD47 isoforms in conditioned medium (Figure 9a). Activation of the BJAB EBV-negative B cell line and the EBV-positive RAJI B cell line increased the expression of high-molecular-weight CD47 isoforms that were smaller than those produced by T cells, in addition to expressing the standard ~50 kDa N-glycosylated isoform of CD47.

Activation induced the expected increase in cell surface CD69 in BJAB cells, but, consistent with T cells, activation had minimal effects on the level of CD47 on the surface (Figure 9b and Supplementary Materials). Although activation can increase CD69 expression in some RAJI cells [66], cell surface CD69 was high basally in the RAJI cells used and neither CD69 nor CD47 changed following activation (Figure 9b). Nanoflow analysis indicated that the BJAB and RAJI B cell lines release CD47^+^ EVs larger than 35 nm and that CD47 was associated with smaller particles (Figure 9c). As seen in T cells, induction of glycosaminoglycan biosynthesis may occur during B cell activation because a search of the published data identified increased mRNA expression for EXT1, CHST11, and HS2ST1 when mouse splenic B cells were activated by treatment for 4 h with anti-IgM or anti-CD40 (GEO Dataset: GDS5428, [67]).

## 3. Discussion

We previously found that T cells constitutively express proteoglycan isoforms of APLP2 and CD47 that bear both heparan and chondroitin sulfate chains [42]. Although thrombospondin-1 binds to both cell surface proteoglycans in vitro, only CD47 mediates inhibition of T cell receptor signaling by thrombospondin-1. Glycosaminoglycan modification of CD47 at Ser^64^ is required for this inhibitory signaling [42]. The present study revealed that the glycosaminoglycan modification of these T cell surface proteoglycans is remodeled during activation. Sulfate incorporation into T cell heparan sulfate and chondroitin sulfate glycosaminoglycans that modify APLP2 and CD47 is induced following T cell activation. However, most of this highly glycosylated CD47 and APLP2 is released into the medium, while the T cells maintain relatively constant cell surface levels of CD47. The homeostasis of cell surface CD47 density may be specific, because cell surface APLP2 levels increased under the same activation conditions.

Although we cannot exclude contributions of proteolytic shedding to the release of both proteins induced by activation, significant fractions of the released CD47 and APLP2 appear to be intact and are associated with EVs. Similar induction of CD47 release following B cell receptor engagement was also observed for two cell lines of B cell lineage, suggesting that this is a general feature of lymphocyte activation. These CD47^+^ EVs may contribute to intercellular communication regulating immune responses because the CD47^+^ EVs released by T cells contain a distinct population of small RNAs and have specific effects on gene expression in cells that take up these EVs [53,56]. The presence of isoforms of CD47 that bind thrombospondin-1 with high affinity on these EVs may also serve as a sponge to adsorb thrombospondin-1 and thereby limit its immunosuppressive activity at sites of T cell activation. Others have reported that CD47^+^ EVs can associate with the surfaces of target cells in a manner that does not contribute to cell signaling but protects those target cells from phagocytic clearance [68]. Further study is needed to define how the CD47^+^ EVs produced by activated B and T lymphocyte lineages regulate intercellular communication relevant to innate and adaptive immune responses in cancer and infectious diseases.

We observed a net shift from heparan to chondroitin sulfate modification of CD47 upon T cell activation. This may decrease the sensitivity to CD47-mediated inhibition of T cell receptor signaling by thrombospondin-1 because prior studies have shown that thrombospondin-1 binds selectively to heparan sulfate [69]. These data suggest a negative feedback loop by which thrombospondin-1 controls the level of a post-translational modification of its own receptor that is required to transduce inhibitory thrombospondin-1 signals in T cells.

The extracellular domain of CD47 was previously shown to be shed from the surface of vascular smooth muscle cells via proteolytic cleavage, which was suppressed under conditions of hyperglycemia [51]. Our studies indicate that adaptive immune cells can also release intact CD47 on EVs. CD47 on EVs may also serve as a decoy to block signaling through engaging CD47 on T cells. Engraftment studies have shown that the expression of CD47 on T cells is important for their homeostasis [70]. This function was attributed to SIRPα interactions [70,71], but the shed extracellular domain of the proteoglycan isoform of CD47 can also bind thrombospondin-1 and thus may modulate its known effects on T cell homeostasis under inflammatory conditions [41]. Because CD47 was previously implicated in mediating uptake of thrombospondin-1 by T cells [72], the presence of CD47 isoforms that bind thrombospondin-1 on EVs could also be viewed as a mechanism to limit T cell clearance of thrombospondin-1 from the extracellular space. Further research is needed to define the regulation of CD47 release from immune cells and to characterize the biological activities of shed proteoglycan isoforms of CD47 either alone or in complex with thrombospondin-1.

Future studies are needed to examine the relevance of the activation-induced release of CD47 associated with EVs on immune phenotypes that have been reported in *Cd47*^−/−^ mice. Healthy *Cd47*^−/−^ mice generally exhibit normal immune function, but differences emerge when challenged with specific pathogens [61,73,74,75,76,77]. Lower numbers of splenic CD4^+^ T cells that may depend on CD47 functions in dendritic cells were reported [36]. CD8 T cells from *Cd47*^−/−^ mice exhibit enhanced cell proliferation and effector phenotypes following acute activation, but their responses to chronic stimulation are impaired [61]. This is consistent with the observed impairment of CD8 effector function in chronically LCMV-infected *Cd47*^−/−^ mice or mice bearing B16 melanoma tumors [61].

The role of CD47 in B cells is less studied. CD47 is highly expressed on plasma cells in bone marrow and limits antibody-mediated phagocytosis [78]. Protective IgG, induced following influenza viral infection or vaccination, was elevated in *Cd47*^−/−^ mice [79]. Conversely, *Cd47*^−/−^ mice exhibited reduced IgG autoantibody production in the Fas(lpr) autoimmune nephritis model [80]. The functions of CD47 on autoreactive B cells and how interacting T cells drive autoimmune diseases such as systemic lupus erythematous and rheumatoid arthritis [81] merit further study, including investigation of the function of CD47 on EVs released by these activated immune cells.

Multiple humanized CD47 antibodies have been developed, and several have entered clinical trials as agents to enhance innate antitumor immunity [82]; however, the potential direct effects of these antibodies on adaptive antitumor immunity remain unknown. These antibodies were designed to bind to the protein core of CD47 in a manner that inhibits its interaction with SIRPα, but it is unclear whether glycosaminoglycan modifications of CD47 would prohibit therapeutic antibody binding. The increased release of CD47 associated with EVs by activated T and B cells in the tumor microenvironment could also create a sink that limits therapeutic CD47 antibody binding to tumor cells and merits further study.

## 4. Materials and Methods

### 4.1. Cell Culture and Reagents

Wild-type (Clone E6-1, TIB-152™ American Type Culture Collection) and CD47-deficient (Clone JinB8) Jurkat T cells [60] were cultured in RPM1 1640 medium containing 10% FBS, glutamine, and penicillin/streptomycin (Invitrogen) at 37 °C with 5% CO_2_. Primary T cells isolated from the spleens of WT and *Cd47^−/−^* mice and human peripheral blood were cultured using the same medium on plates coated with the indicated antibodies and proteins. The B cell lines BJAB and RAJI cells were provided by Dr. Laurie Krug (NCI) and cultured in TheraPEAK^®^ X-VIVO^®^ 15 Serum-free Hematopoietic Cell Medium (Lonza, Basel, Switzerland). The following antibodies were used for western blots and flow cytometry: anti-human CD47 (B6H12 (Santa Cruz Biotechnologies, Santa Cruz, CA, USA, Abcam, Cambridge, UK, and CD47-APC or PE (Biolegend, San Diego, CA, USA). The APLP2 antibody was from Calbiochem/EMB Chemicals (Cat. No. 171617). Purified anti-human CD3 and murine CD3 antibodies were from BD Pharmingen. Thrombospondin-1 was purified from human platelets obtained from the NIH Blood Bank, Division of Transfusion Medicine, as previously described [83].

### 4.2. Primary T Cell Isolation

Human buffy coats were obtained from healthy volunteer blood donors as byproducts of allogeneic blood donation. Blood donors provided written informed consent use for the use of their blood for research purposes and blood samples were de-identified prior to distribution by the NIH Blood Bank under an approved protocol [42]. Purification of CD4 and CD8 T cells was performed using EasySep™ Human CD4+ and CD8+ T Cell Isolation Kits (Stemcell Technologies, Vancouver, BC, Canada). WT and CD47 null C57Bl/6 mice (B6.129S7-Cd47tm1Fpl/J; Strain:003173, Jackson Laboratories, Bar Harbor, ME, USA) were bred in an AALAC-compliant facility under an approved protocol of the Animal Care and Use Committee of the NCI. Spleens from age- and gender-matched mice aged 6–10 weeks were dissected, and a single cell suspension was prepared using dissociation buffer (PBS pH7.2, 0.5% BSA and 2 mM EDTA). The cells were filtered with 40 µm nylon mesh filters to remove cell clumps. The single-cell suspension was filtered, and T cells were isolated using the Pan-T cell isolation kit (Miltenyi Biotech, Auburn, CA, USA). For activation studies, plates were coated overnight using anti-mouse or anti-human CD3 antibody (1–5 µg/mL as indicated). The coated plates were washed three times with PBS before being used for cell culture. The q-PCR analysis was performed as described earlier [42], and fold change in mRNA expression was calculated normalized to B2M mRNA levels. SD represents two technical replicates.

### 4.3. Proteoglycan and Glycosaminoglycan Analysis

Metabolic labeling of primary human peripheral blood T cells and Jurkat cells with ^35^S-sulfate was performed as previously described [42]. Labeling was performed using resting cells or cells stimulated using PMA or immobilized anti-human CD3 antibody. Metabolically labeled proteoglycans were isolated and analyzed as described [42].

^3^H/^35^S-labeled proteoglycan fractions were isolated from Jurkat cell-conditioned medium, essentially as described previously using modified HITES medium (90% F-12K Nutrient Mixture, Kaighn’s Modification (Gibco, New York, NY, USA), 10% RPMI 1640 (Gibco), 5 mM HEPES, 2 mM L-glutamine, 0.1% BSA (tissue culture grade Sigma), 1x Insulin-Transferrin-Selenium-A Supplement (Gibco), and 200 nM hydrocortisone (Biofluids, Los Osos, CA, USA), supplemented with [6-^3^H]-glucosamine plus [^35^S]-sulfuric acid at final concentrations of 20 µCi/mL and 100 µCi/mL, respectively (Perkin-Elmer, Waltham, MA, USA).

The radiolabeled Jurkat cells were divided in half and placed into two T175 Nunc tissue culture flasks that had been pre-incubated with or without 10 µg/mL of anti-CD3 stimulating antibody. Following overnight incubation at 37° in 5% CO_2_, conditioned media from each flask was separated from the cells by centrifugation and aliquoted into two equal volumes and stored in labeled 50 mL tubes.

Conditioned medium was prepared for ion-exchange chromatography by addition of 8 M urea and 0.15 M NaCl, and the pH was adjusted to 4.5 using acetic acid. Conditioned media were applied to Q-Sepharose fast flow columns equilibrated in 8 M urea, 0.05 M sodium acetate, pH 6.0, and eluted either using a step gradient with 1 M NaCl or with linear NaCl gradients from 0.15 to 1 M. Fractions were analyzed by scintillation counting and pooled for further use.

For some experiments, the proteoglycan fractions were concentrated by diluting 8-fold in 8 M urea and applying to 0.1 mL columns of Q-Sepharose fast flow. After washing with column buffer, the proteoglycan was eluted by a step gradient to 1 M NaCl in column buffer. Purified ^35^S-labeled proteoglycan fractions were digested with chondroitinase ABC or heparitinase III as previously described [42].

BJAB and RAJI cells were grown overnight in X-VIVO medium in the presence or absence of anti-IgM (2 µg/mL). Conditioned media from control and treated cells were immunoprecipitated using B6H12 anti-CD47 magnetic beads and analyzed using western blotting.

### 4.4. Extracellular Vesicle Extraction and Metabolic Labelling

EVs were isolated and characterized following recommended guidelines [62]. Jurkat and human T cells were cultured using HITES media with or without radiolabeling with ^35^S-sulfate on CD3-antibody-coated plates for times indicated on the western blot analysis. A 50 mL volume of each conditioned medium was concentrated to 0.05 mL by ultrafiltration and applied onto qEV70 columns. The EV fractions were pooled and analyzed by immunoblotting using APLP2 and CD47 antibodies.

BJAB and RAJI cells were cultured in X-VIVO medium for 48 h. A total of 50 mL of conditioned media was harvested, concentrated to 0.5 mL by ultrafiltration, and applied onto qEV35 columns; 15 fractions were collected. Fractions 5–9 (EVs) and 10–15 were pooled, diluted 1:2 with filtered 1X PBS, stained with -PE-conjugated CD47 antibody (Biolegend), and data were acquired using at a Beckman Coulter CytoFlex Nano. Extracellular vesicles were identified using VSSC1-H vs. FSC-H with a 405 nm laser and 405/10 filter. The anti-CD47 signal was detected with 561 nm excitation and a 595/50 filter using the height parameter.

## Figures and Tables

**Table 1:** Effect of anti-CD3 + CD28 activation on mRNA expression of glycosaminoglycan biosynthetic genes in mouse CD8 T cells. Differentially expressed genes with *p*-values < 0.001 were identified from RNA sequencing data (Data Supplement S1 in [61]) (available at the Gene Expression Omnibus (GEO) database under accession number GSE198820).

Gene	CD3+CD28 vs. Untreated WT CD8 T Cells (Fold)	*p*-Value	CD3+CD28 vs. Untreated *Cd47^−/−^* CD8 T Cells (Fold)	*p*-Value
Ext1	-	NS *	-	NS
Ext2	-	NS	-	NS
Ndst1	-	NS	-	NS
Chsy1	1.89	3.2 × 10^−7^	1.50	8.2 × 10^−5^
Chsy3	-	NS	-	NS
Chpf	2.56	6.0 × 10^−8^	2.31	5.4 × 10^−7^
Chst3	−2.53	3.3 × 10^−4^	-	NS
Chst11	2.04	1.8 × 10^−4^	-	NS
Glce	1.70	5.0 × 10^−7^	1.52	1.4 × 10^−5^
Extl2	2.72	6.0 × 10^−7^	-	NS
Extl3		NS		NS
Hs2st1	-	NS	-	NS
Hs6st1	-	NS	-	NS

* not significant = differential expression *p* > 0.001.

**Table 2:** Effect of Jurkat cell activation on glycosaminoglycan biosynthesis and sulfation. Unstimulated Jurkat cells and cells plated on immobilized anti-CD3 were metabolically labeled with ^3^H-glucosamine and ^35^S-sulfate for 24 h. Proteoglycans were isolated from conditioned medium by ion-exchange chromatography on Q-Sepharose and analyzed by re-chromatography with gradient elution without digestion or after digestion with heparitinase or chondroitinase ABC. Incorporation into the proteoglycan peak fractions (35–68) was quantified by scintillation counting.

Treatment	Σ(^3^H dpm)	Σ(^35^S dpm)	Ratio ^35^S/^3^H
Undigested	2500	628	0.25
Heparitinase digest	1560	436	0.27
Chondroitinase digest	1260	182	0.14
Anti-CD3-activated/Undigested	22,980	4100	0.17
Anti-CD3/Heparitinase digest	12,800	2820	0.21
Anti-CD3/Chondroitinase digest	9700	1380	0.14

## Data Availability

Data supporting the reported results can be found in the Supplementary Materials and links to the indicated publicly archived datasets analyzed or generated during the study.

## Supplementary Materials

The following supporting information can be downloaded at: https://www.mdpi.com/article/10.3390/ijms26178377/s1.

## Abbreviations

The following abbreviations are used in this manuscript:

APLP2	Amyloid precursor-like protein-2
CHPF	Chondroitin polymerizing factor
CHST	Carbohydrate sulfotransferase
CHSY	Chondroitin sulfate synthase
EVs	Extracellular vesicles
EXT	Exostosin glycosyltransferase
HSPG	Heparan sulfate proteoglycan
NDST	N-deacetylase/N-sulfotransferase (heparan glucosaminyl)
PMA	Phorbol *12-*myristate *13-*acetate
SDS	Sodium dodecyl sulfate
SIRPα	Signal regulatory protein-α
TSP1	Thrombospondin-1
