# Supplementary material for: T Cell Activation Induces Synthesis of CD47 Proteoglycan Isoforms and Their Release in Extracellular Vesicles
Source: Int J Mol Sci. 2025 Aug 28;26(17):8377. doi: 10.3390/ijms26178377 (PMC12428540; doi:10.3390/ijms26178377)
Supplement: Supplementary file 1 [file ijms-26-08377-s001.zip › B cell activation _MFI_CD47_CD69_BJAB_RAJI.pptx]

## Slide 1
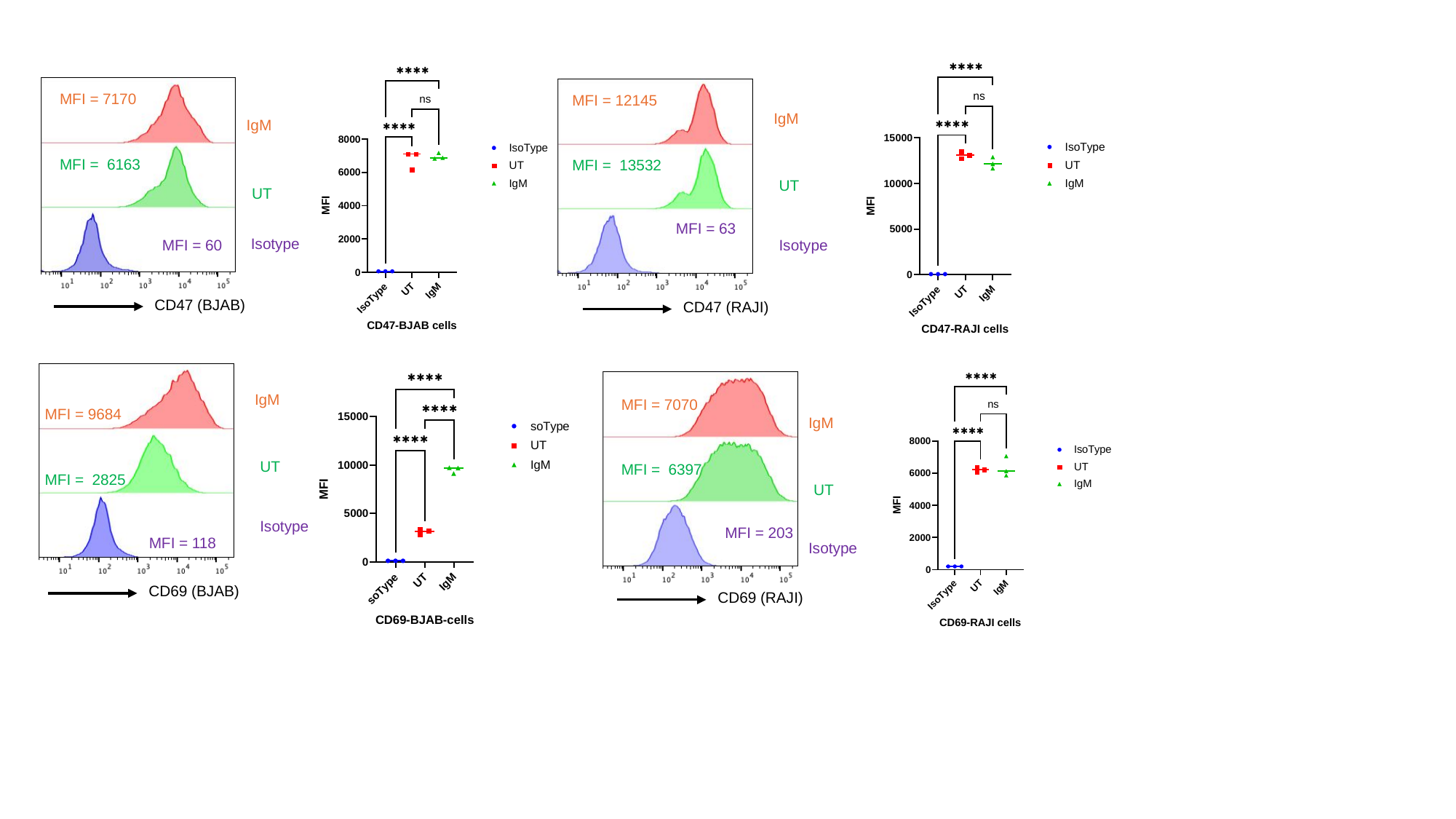

MFI = 7170
MFI = 12145
IgM
IgM
MFI = 6163
MFI = 13532
UT
UT
MFI = 63
Isotype
MFI = 60
Isotype
CD47 (BJAB)
CD47 (RAJI)
IgM
MFI = 7070
MFI = 9684
IgM
UT
MFI = 6397
MFI = 2825
UT
Isotype
MFI = 203
MFI = 118
Isotype
CD69 (BJAB)
CD69 (RAJI)
