# Supplementary material for: T Cell Activation Induces Synthesis of CD47 Proteoglycan Isoforms and Their Release in Extracellular Vesicles
Source: Int J Mol Sci. 2025 Aug 28;26(17):8377. doi: 10.3390/ijms26178377 (PMC12428540; doi:10.3390/ijms26178377)
Supplement: Supplementary file 1 [file ijms-26-08377-s001.zip › CD47 & CD69-Final Figure.pptx]

## Slide 1
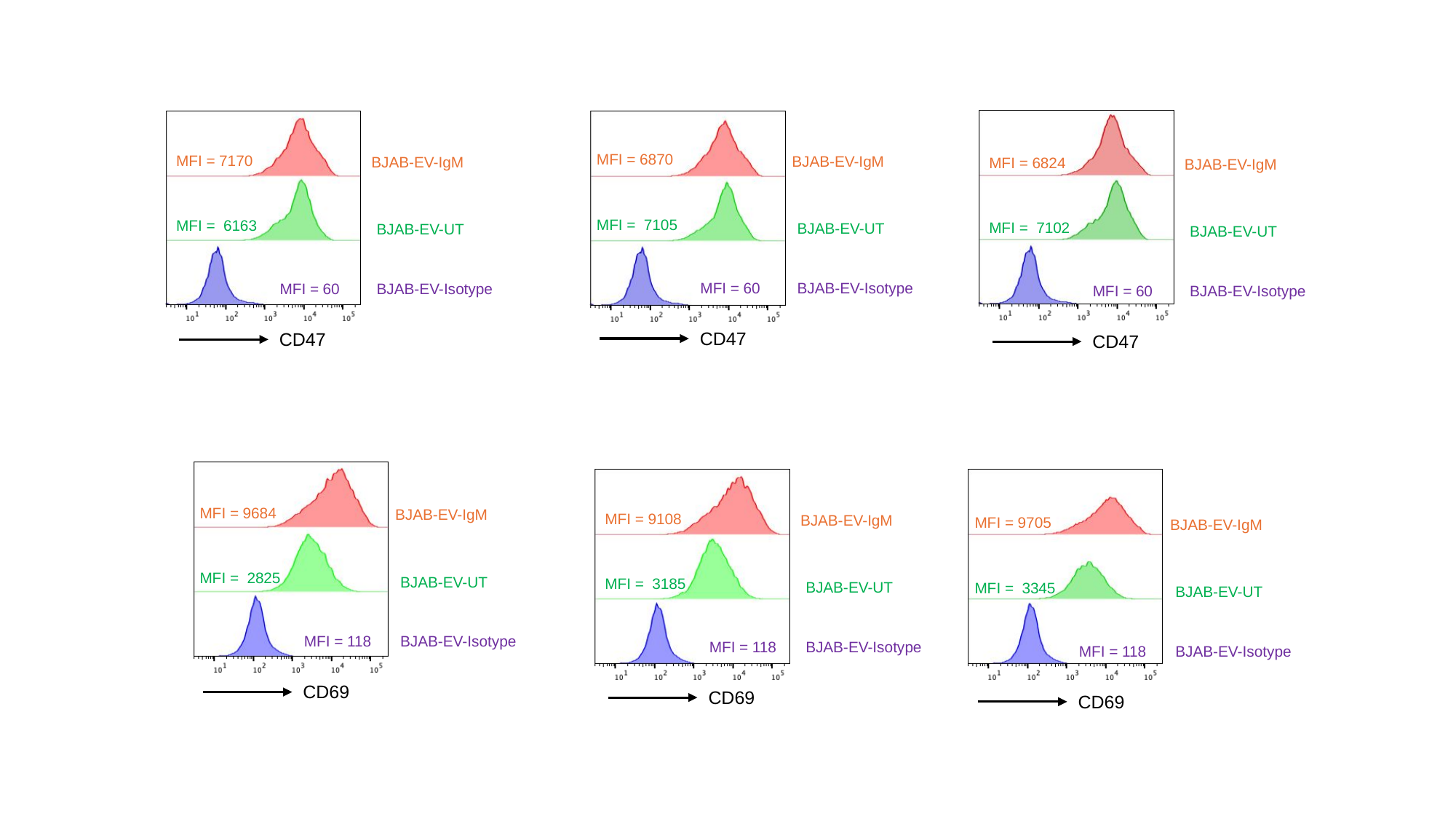

MFI = 6870
MFI = 7170
BJAB-EV-IgM
BJAB-EV-IgM
MFI = 6824
BJAB-EV-IgM
MFI = 7105
MFI = 6163
MFI = 7102
BJAB-EV-UT
BJAB-EV-UT
BJAB-EV-UT
MFI = 60
BJAB-EV-Isotype
MFI = 60
BJAB-EV-Isotype
MFI = 60
BJAB-EV-Isotype
CD47
CD47
CD47
MFI = 9684
BJAB-EV-IgM
MFI = 9108
BJAB-EV-IgM
MFI = 9705
BJAB-EV-IgM
MFI = 2825
BJAB-EV-UT
MFI = 3185
BJAB-EV-UT
MFI = 3345
BJAB-EV-UT
MFI = 118
BJAB-EV-Isotype
MFI = 118
BJAB-EV-Isotype
MFI = 118
BJAB-EV-Isotype
CD69
CD69
CD69
