# Supplementary material for: T Cell Activation Induces Synthesis of CD47 Proteoglycan Isoforms and Their Release in Extracellular Vesicles
Source: Int J Mol Sci. 2025 Aug 28;26(17):8377. doi: 10.3390/ijms26178377 (PMC12428540; doi:10.3390/ijms26178377)
Supplement: Supplementary file 1 [file ijms-26-08377-s001.zip › Comp(CD47).pptx]

## Slide 1
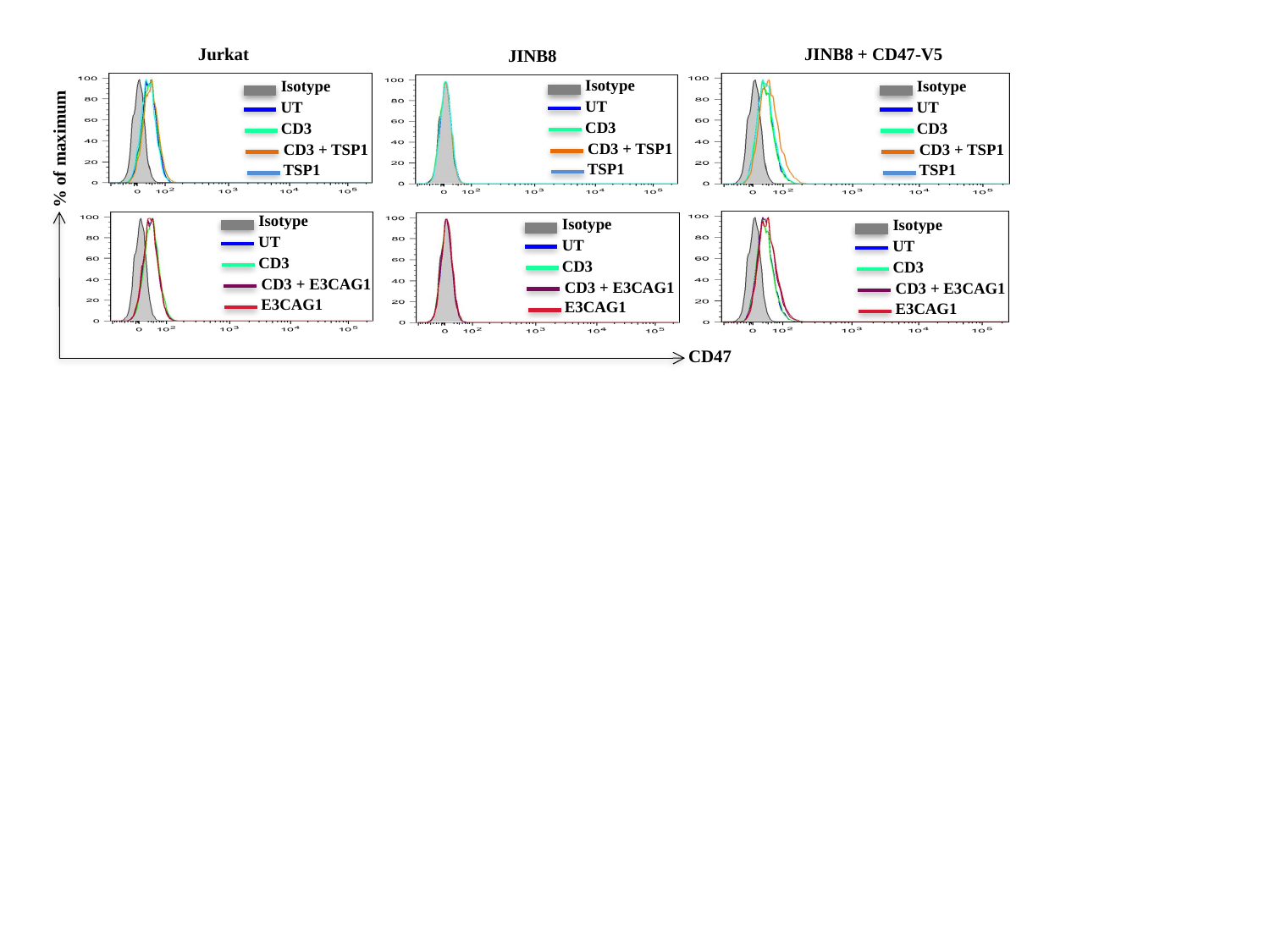

JINB8 + CD47-V5
Jurkat
JINB8
Isotype
UT
CD3
CD3 + TSP1
TSP1
Isotype
UT
CD3
CD3 + TSP1
TSP1
Isotype
UT
CD3
CD3 + TSP1
TSP1
% of maximum
Isotype
UT
CD3
CD3 + E3CAG1
E3CAG1
Isotype
UT
CD3
CD3 + E3CAG1
E3CAG1
Isotype
UT
CD3
CD3 + E3CAG1
E3CAG1
CD47
