# Supplementary material for: T Cell Activation Induces Synthesis of CD47 Proteoglycan Isoforms and Their Release in Extracellular Vesicles
Source: Int J Mol Sci. 2025 Aug 28;26(17):8377. doi: 10.3390/ijms26178377 (PMC12428540; doi:10.3390/ijms26178377)
Supplement: Supplementary file 1 [file ijms-26-08377-s001.zip › Comp-Cd47 & CD69-RAJI.pptx]

## Slide 1
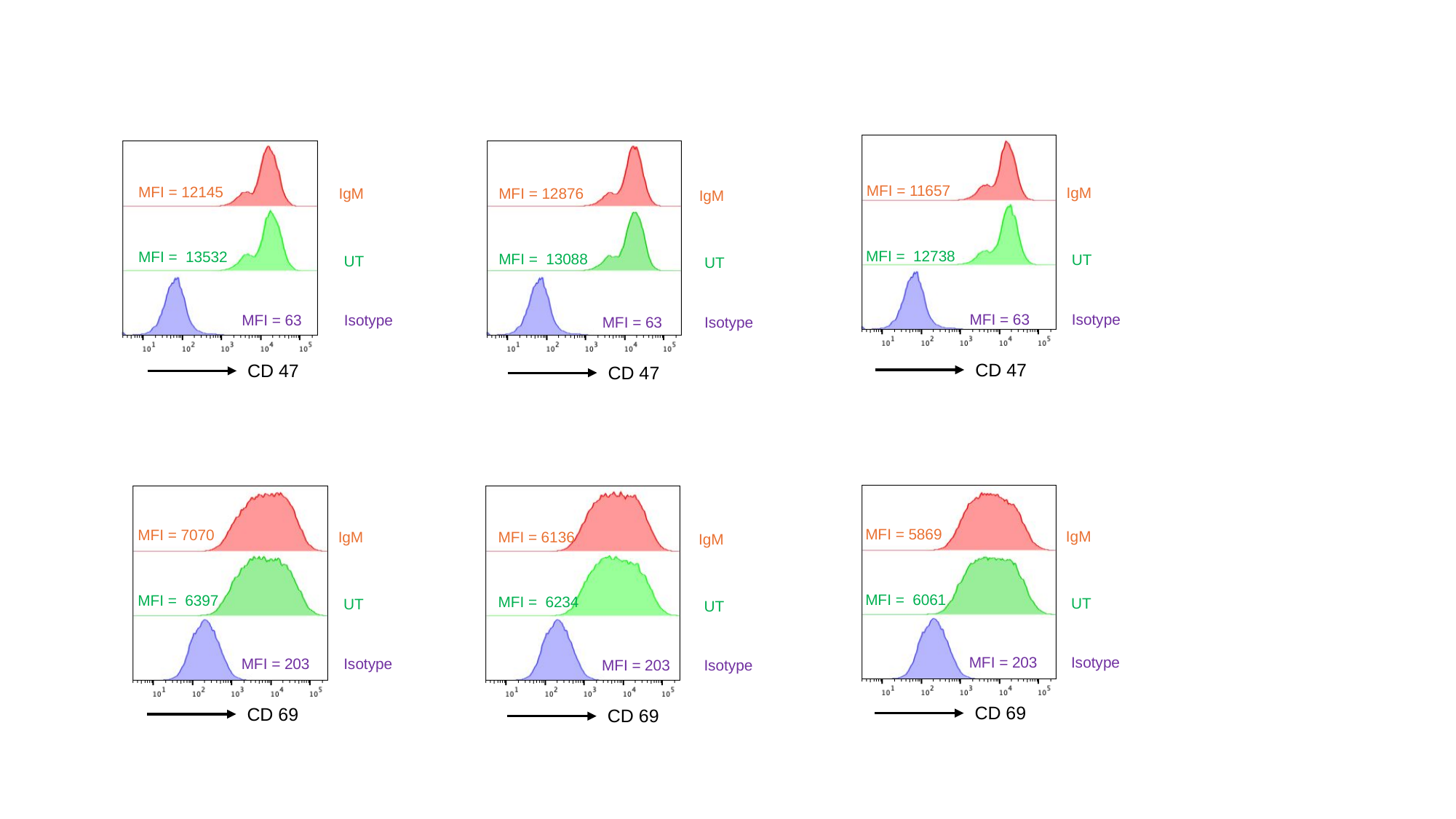

MFI = 11657
MFI = 12145
IgM
IgM
MFI = 12876
IgM
MFI = 12738
MFI = 13532
MFI = 13088
UT
UT
UT
MFI = 63
Isotype
MFI = 63
Isotype
MFI = 63
Isotype
CD 47
CD 47
CD 47
MFI = 5869
MFI = 7070
IgM
IgM
MFI = 6136
IgM
MFI = 6061
MFI = 6397
MFI = 6234
UT
UT
UT
MFI = 203
Isotype
MFI = 203
Isotype
MFI = 203
Isotype
CD 69
CD 69
CD 69
