# Supplementary material for: T Cell Activation Induces Synthesis of CD47 Proteoglycan Isoforms and Their Release in Extracellular Vesicles
Source: Int J Mol Sci. 2025 Aug 28;26(17):8377. doi: 10.3390/ijms26178377 (PMC12428540; doi:10.3390/ijms26178377)
Supplement: Supplementary file 1 [file ijms-26-08377-s001.zip › Comp.pptx]

## Slide 1
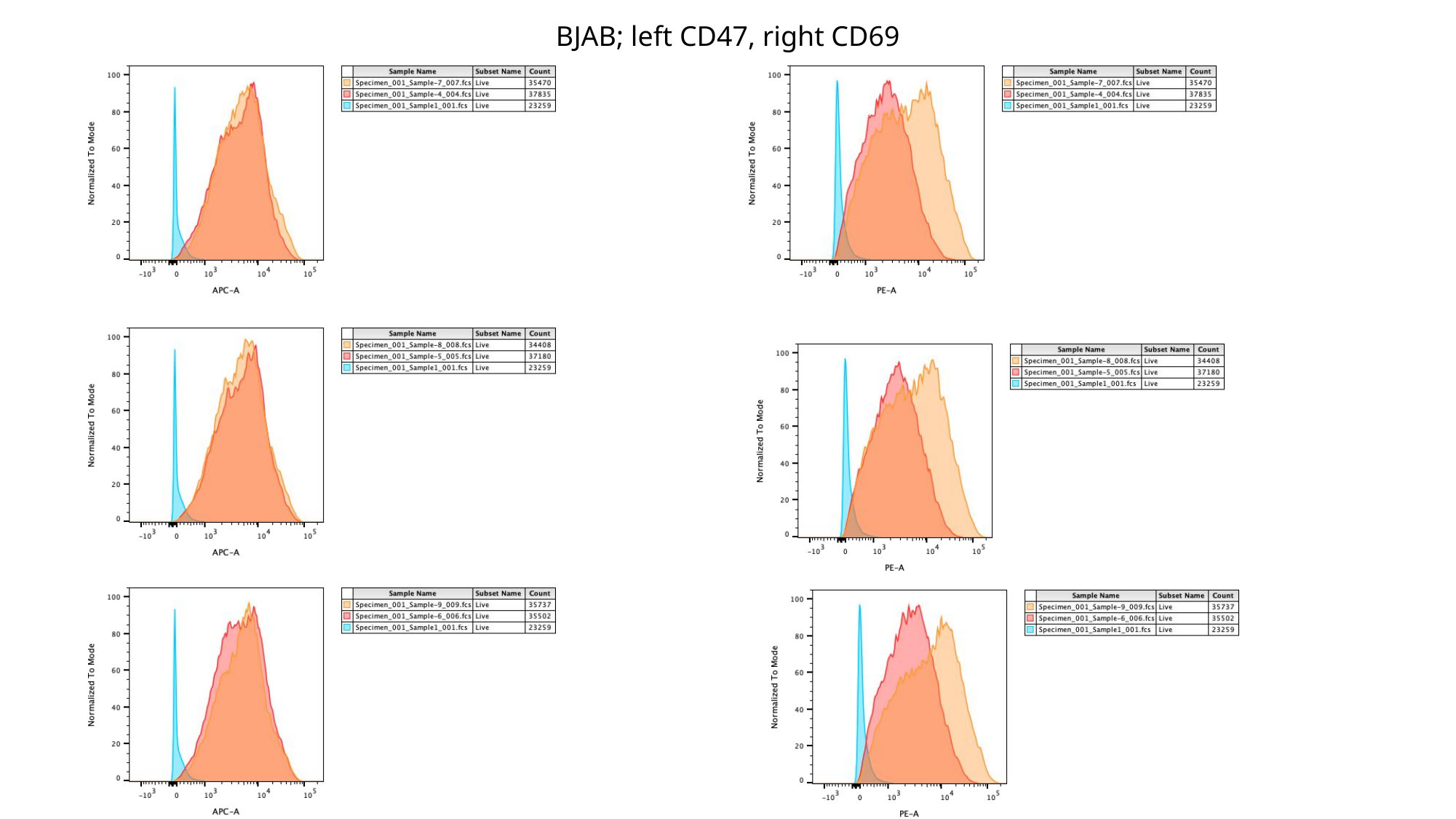

BJAB; left CD47, right CD69

## Slide 2
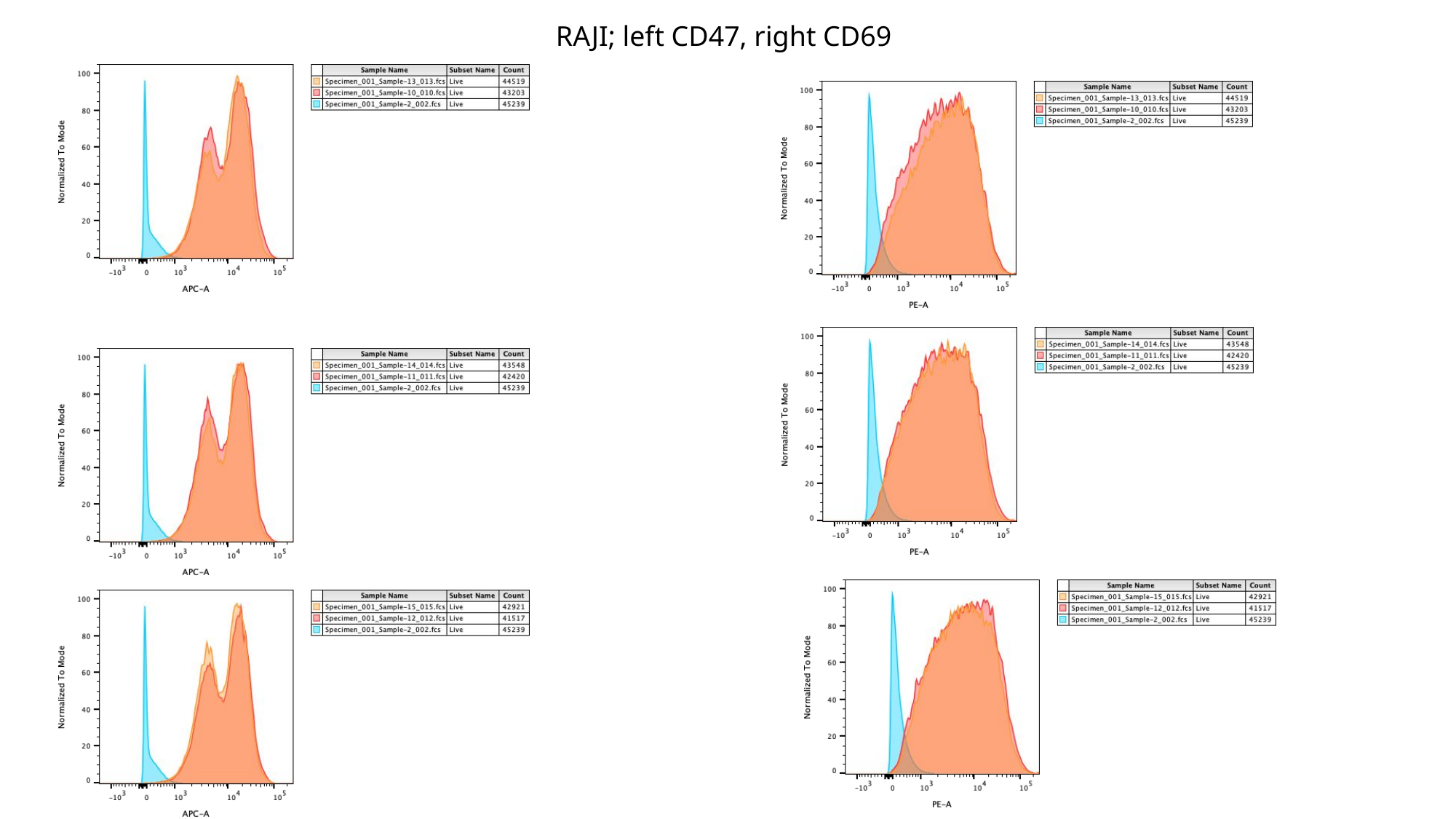

RAJI; left CD47, right CD69

## Slide 3
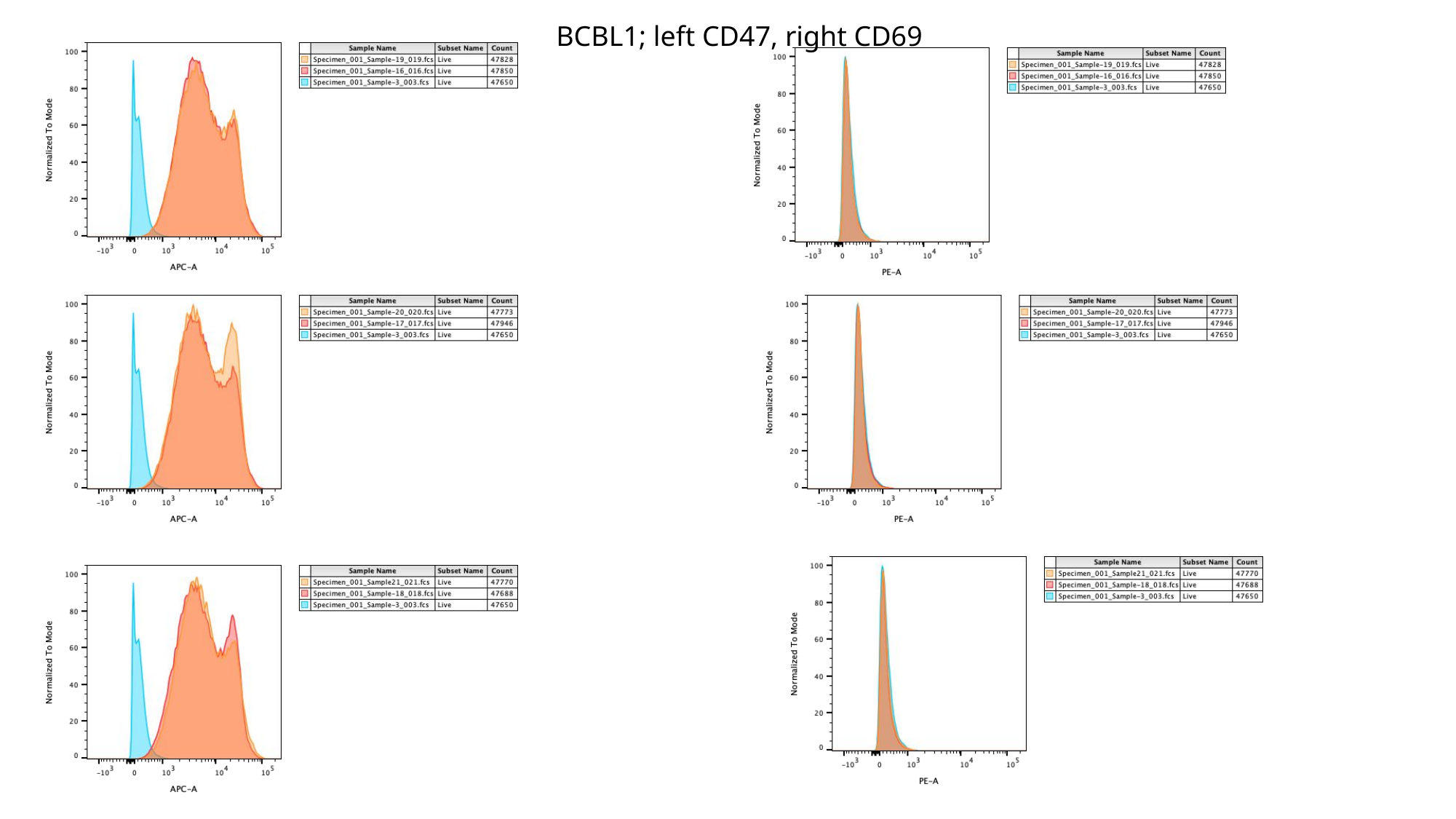

BCBL1; left CD47, right CD69

## Slide 4
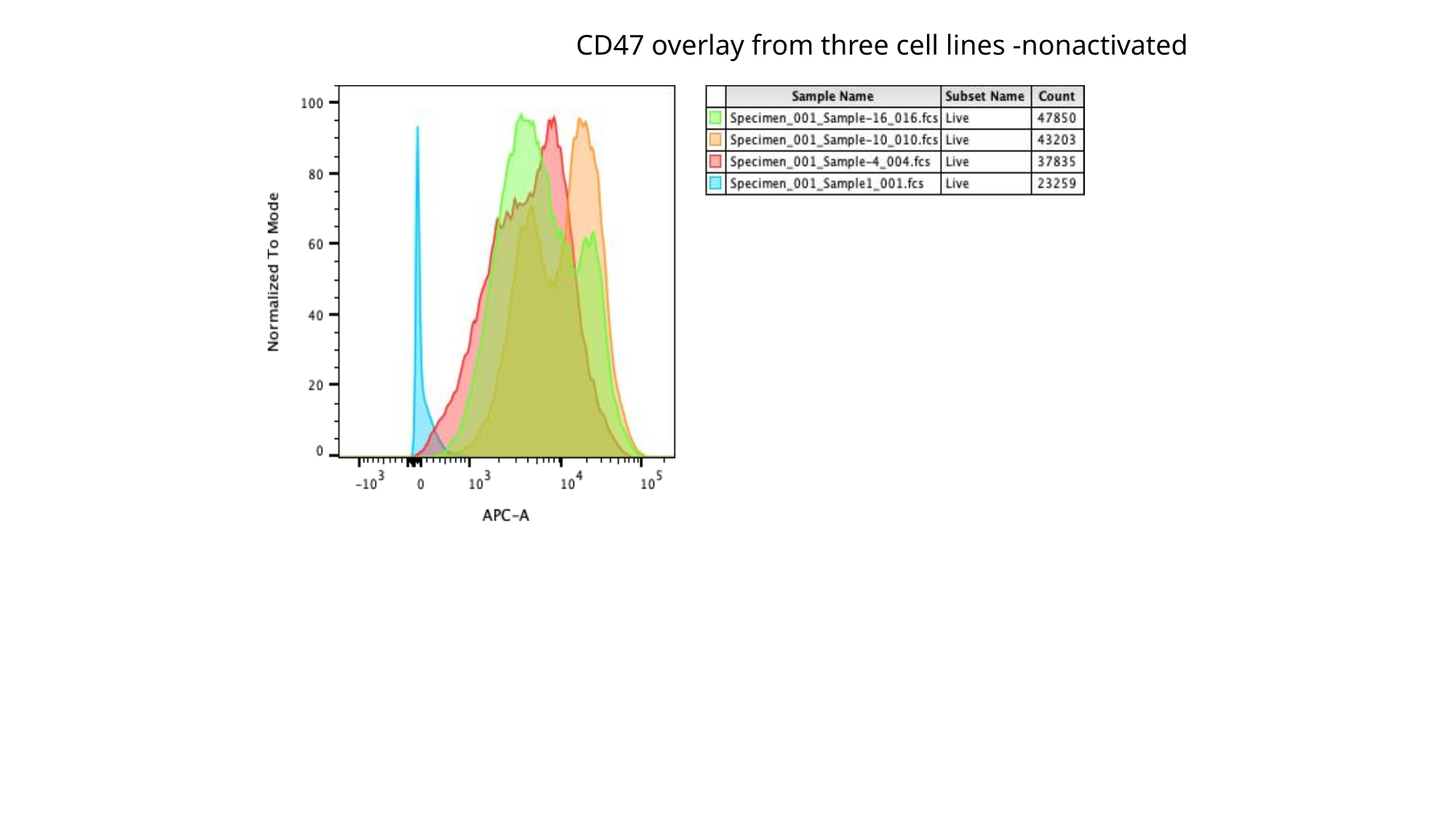

CD47 overlay from three cell lines -nonactivated
