# Supplementary material for: T Cell Activation Induces Synthesis of CD47 Proteoglycan Isoforms and Their Release in Extracellular Vesicles
Source: Int J Mol Sci. 2025 Aug 28;26(17):8377. doi: 10.3390/ijms26178377 (PMC12428540; doi:10.3390/ijms26178377)

Kaur et al Data supplement

Table S1. qPCR Primers (5′ to 3′)

Gene	Primer 1	Primer 2	NCBI Reference
B2M	GAT AGA AAG ACC AGT CCT TGC T	TCC TGA ATT GCT ATG TGT CTG GGT	NG_012920
CHSY3	CAG TTG CAT GGT TGA GGC GA	AAG CCA AGA AGT AGG AGT GGT GCA	NM_175856.4
CHPF	TCA GGT GCT GTT GCC CTG CT	TCA GGT GCT GTT GCC CTG CT	NM_014918.4
CHST11	GTA CAG CTG CGT TTG GTG CT	GCA GCT ACC TGA AGT TCC CCA CC	NM_018413
CHST3	GTT TGT AGC CGA AGA GGC GCA	GCA GTT CGA GAA GTG GCG CT	NM_004273
CHPF2	CTA GCC CCT CCA GGT TGC TG	GTA GAG CCA GGG CTG GTG CAG A	NM_019015
SULF1	CGA GGC ATT TTG AAT CAG CTA CAC	AGG TCA TAG CTT CCT CCA TCT	NM_001128205
SULF2	ACA AGC AGT GTA ACC CCC GGA	CTG GCC ACT TTC GAC GCT GAA AC	NM_018837
CHSY1	AGG AGT AGT CCA CGT CCA CC	ACC CAT AGG TCG ATG CTT TGG A	NM_014918
NDST1	TTG GAT TCC CGA GCC TTC CT	CCT CTC GTA GCC AAG TGG GA	NM_001543.4
EXT1	GGT TTG GCT ACA TGC CGC TGA	CAA AGT CGC TCA ATG TCT CGG	NM_000127.2
EXT2	CAG TCT TCG GGA CCA TGC CT	AAG CTG CCA ATG TTG GGG AAG CTC T	NM_000401.3
CSGLCAT	GTA GAG CCA GGG CTG GTG CAG A	CTA GCC CCT CCA GGT TGC TG	NM_019015.1
B2M (Mus)	TGT CCT TCA GCA AGG ACT GG	GGC ATG CTT AAC TCT GCA GGC GT	NM_009735
NDST1 (Mus)	TCC TTT AGG AAG GCT CGG GA	GGG ATT TTG GTG CCA ACT GC	NM_008306.4
CHSY1 (Mus)	TCA GGC TGT CCT GGC AGA GC	CAT TTG GGT CCA CGC AGC AG	NM_001081163.1
CHST11 (Mus)	GGG AAC TTC AGG TAG CCG CTC	ACT ACG ACC TCG TGG GCA AG	NM_021439.2
CHST3 (Mus)	CTC CTC CAG CAG GCT GAT GG	CTG TGG CCC GAC CAT GCA CCT CT	NM_016803.3
CHSY3 (Mus)	CCA GAG TTC AGC CAG TTG CA	CAT TGT GAT CCT AAC TTG GAC CCT	NM_001081328.1
mu NDST1	GGG ATT TTG GTG CCA ACT GC	TCC TTT AGG AAG GCT CGG GA	NM_008306.4

Data supplement for Figure 4
Uncropped fluorogram images for APLP2 and CD47
release into conditioned medium

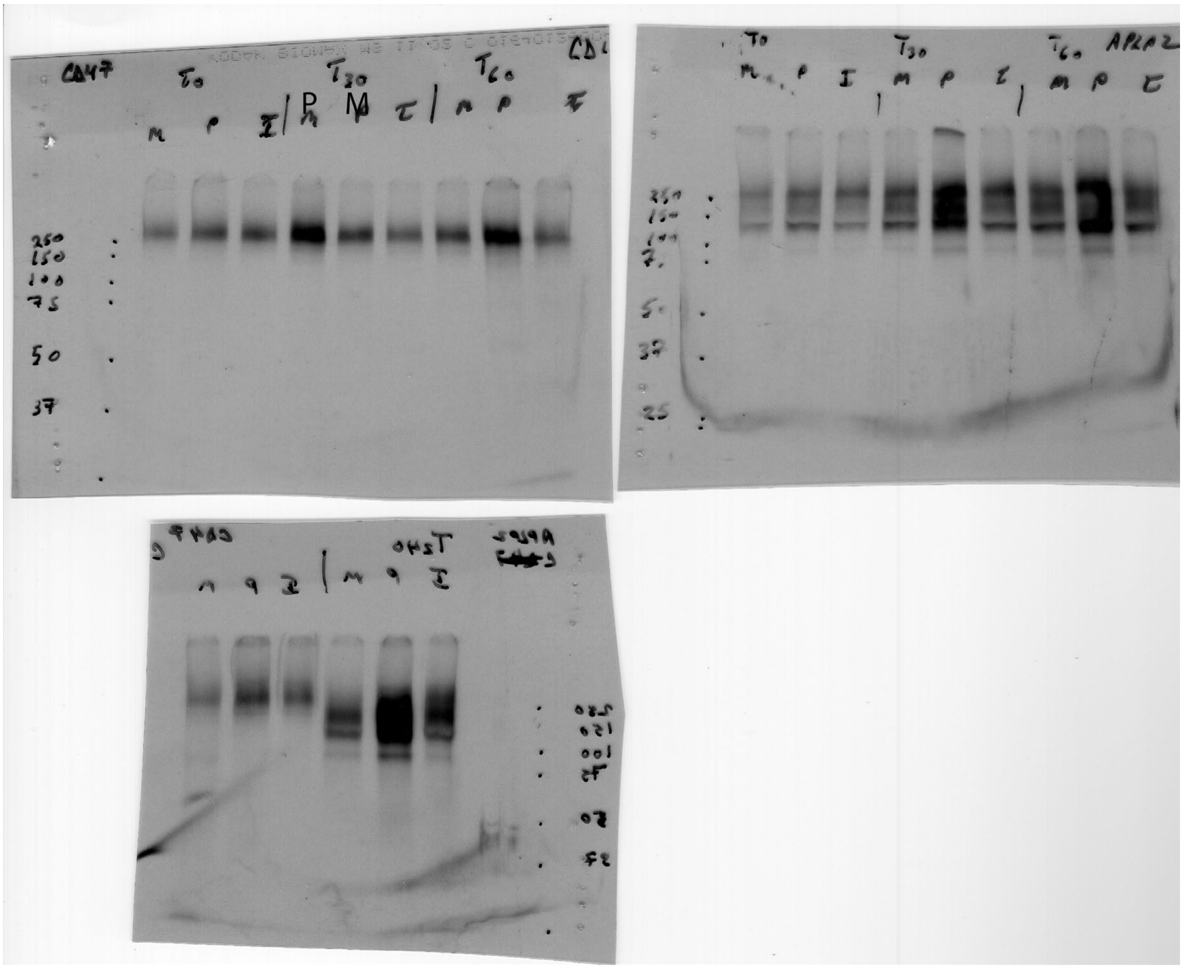

Data supplement for Figure 4
replicate of time course for APLP2 and CD47 release into conditioned medium

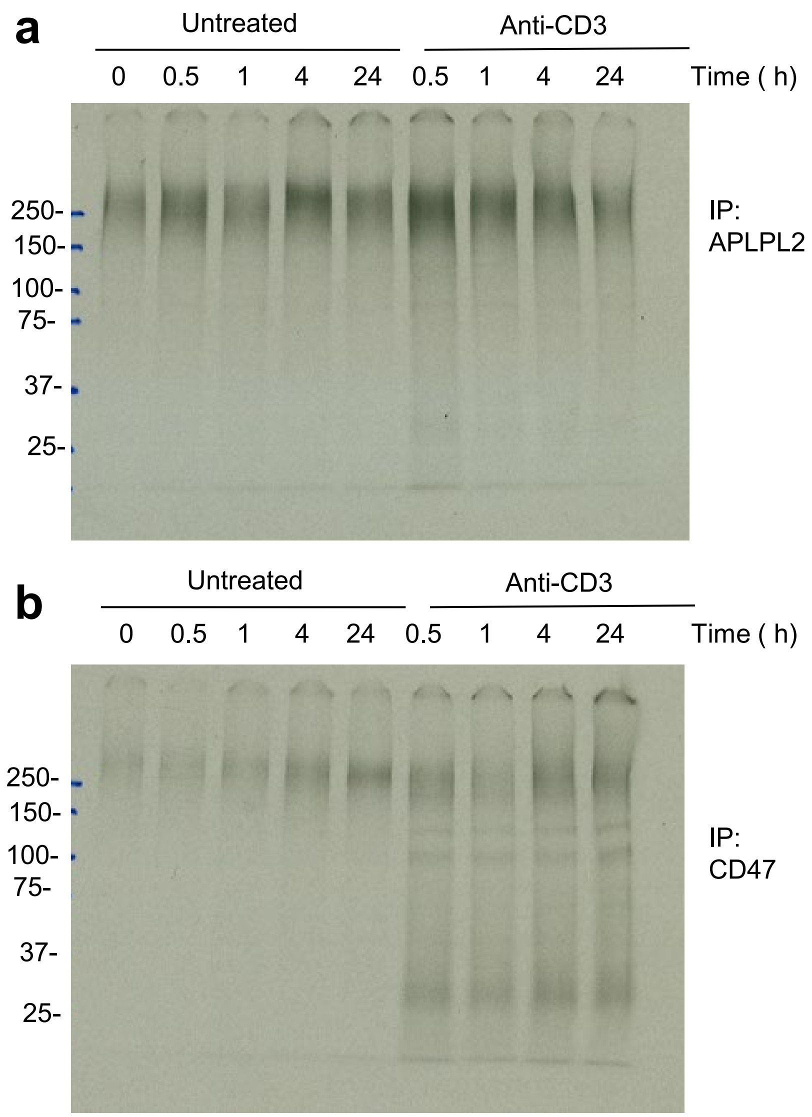

JMS book 2013 p 2 35S conditioned media 1 ug/ml CD3
7.5% BioRad gels

Data supplement for Figure 7
Uncropped fluorogram images for enzymatic digests

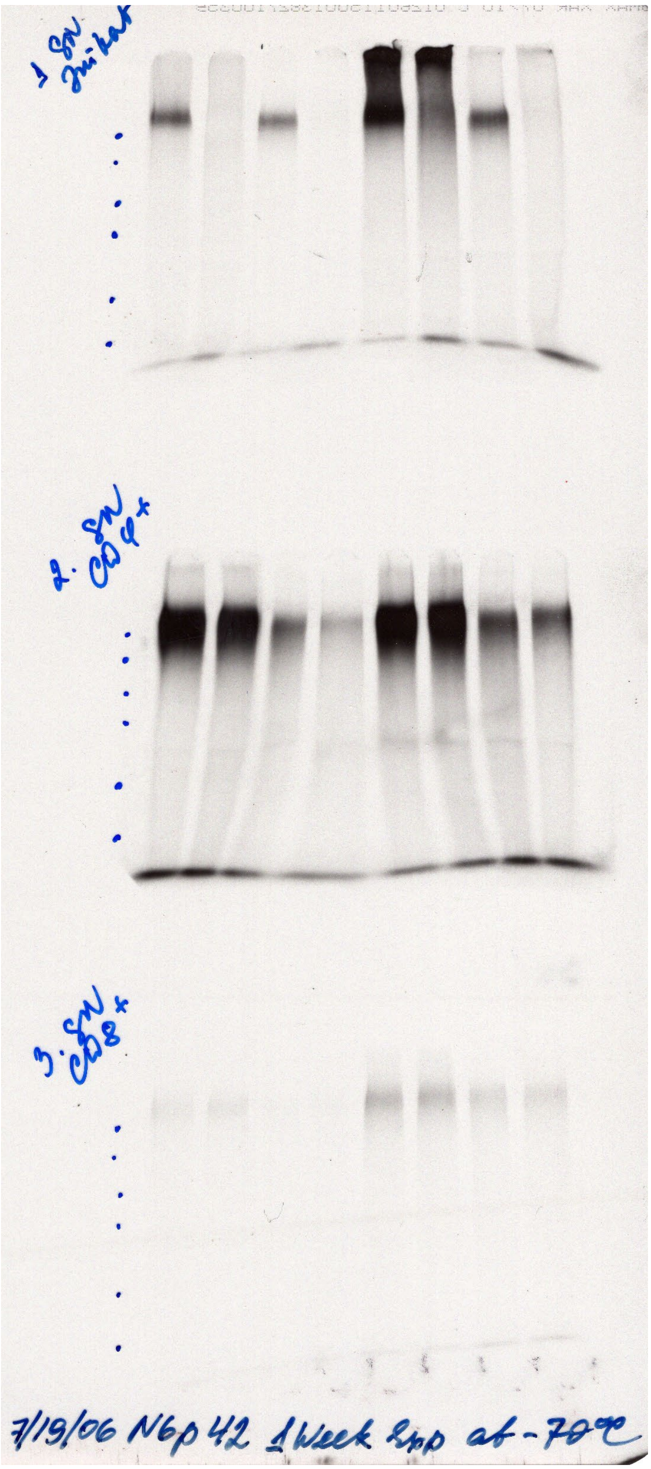

Data supplement for Figure 9

a

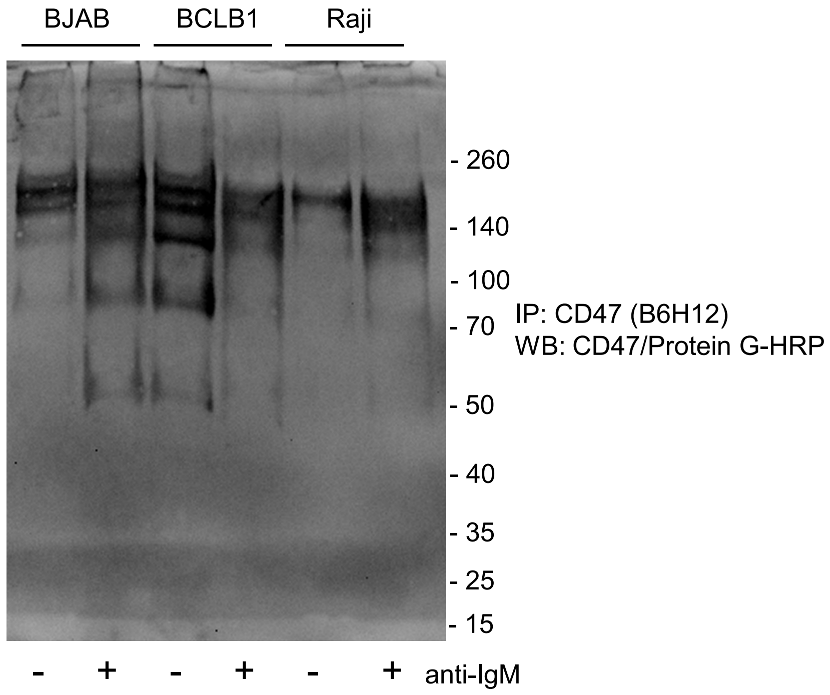

Data supplement for Figure 9
B cell flow cytometry

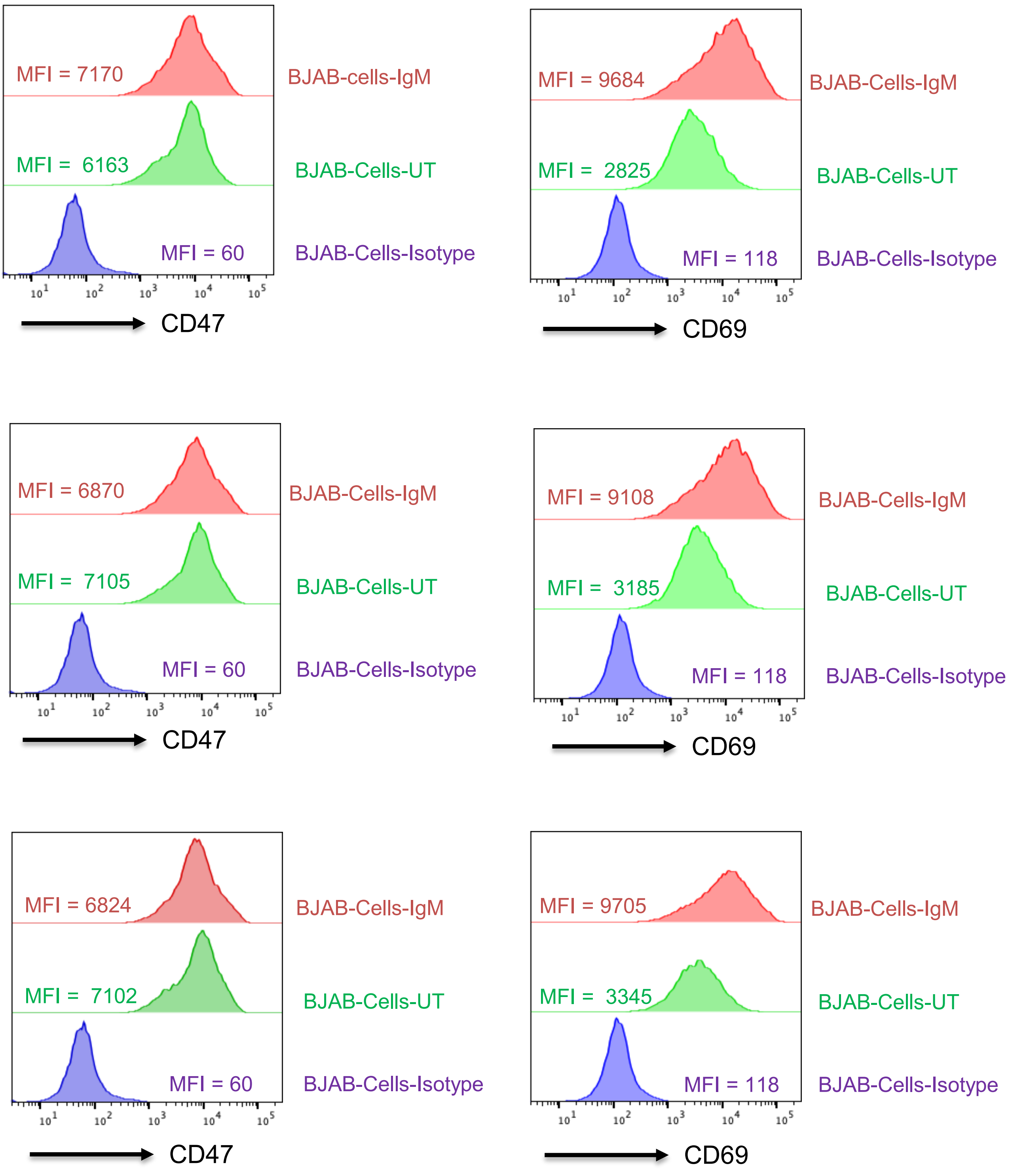

Data supplement for Figure 9 - for RAJI-B cell flow cytometry

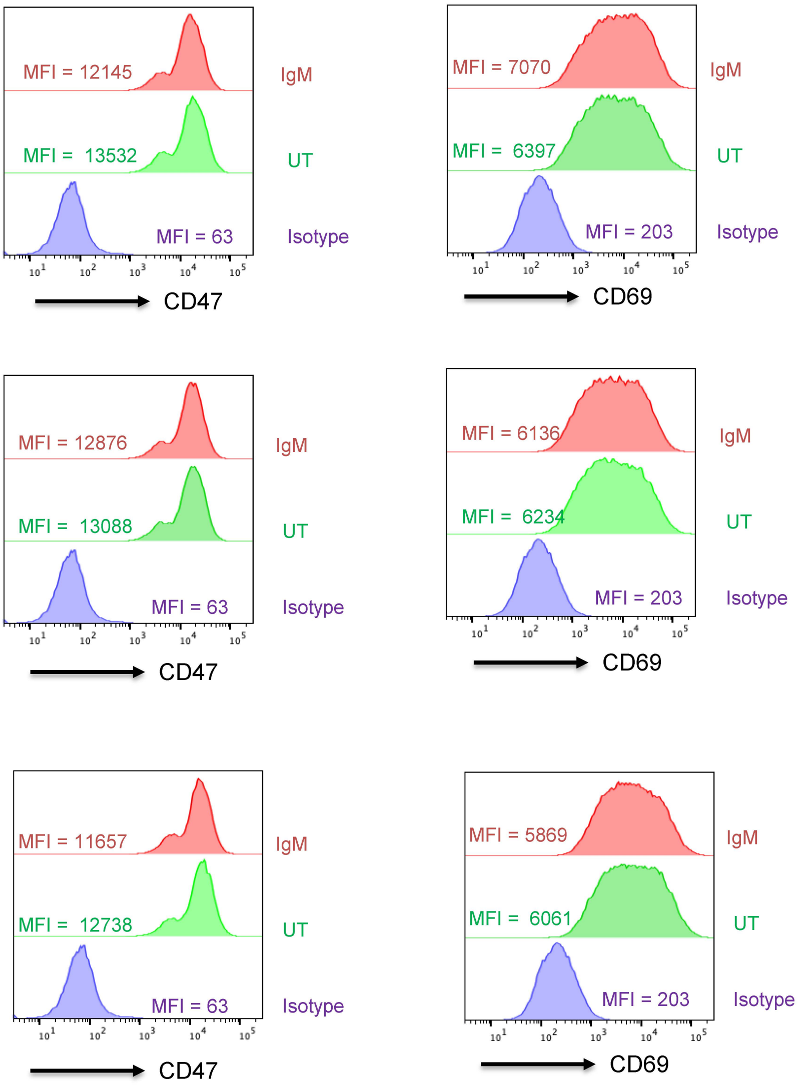

Supplement: Supplementary file 1 [file ijms-26-08377-s001.zip › Kaur et al Data supplement.pdf]
